# Adaptive immunity increases the pace and predictability of evolutionary change in commensal gut bacteria

**DOI:** 10.1038/ncomms9945

**Published:** 2015-11-30

**Authors:** João Barroso-Batista, Jocelyne Demengeot, Isabel Gordo

**Affiliations:** 1 Instituto Gulbenkian de Ciência, Rua da Quinta Grande 6, 2780-156 Oeiras, Portugal

## Abstract

Co-evolution between the mammalian immune system and the gut microbiota is believed to have shaped the microbiota's astonishing diversity. Here we test the corollary hypothesis that the adaptive immune system, directly or indirectly, influences the evolution of commensal species. We compare the evolution of *Escherichia coli* upon colonization of the gut of wild-type and *Rag2*^−/−^ mice, which lack lymphocytes. We show that bacterial adaptation is slower in immune-compromised animals, a phenomenon explained by differences in the action of natural selection within each host. Emerging mutations exhibit strong beneficial effects in healthy hosts but substantial antagonistic pleiotropy in immune-deficient mice. This feature is due to changes in the composition of the gut microbiota, which differs according to the immune status of the host. Our results indicate that the adaptive immune system influences the tempo and predictability of *E. coli* adaptation to the mouse gut.

## Abstract (web summary)

The mechanisms underlying host-commensal coevolution are incompletely understood. Here the authors show that host adaptive immunity directs the evolution of *Escherichia coli* in the mouse gut towards host benefit by influencing the microbiome composition.

## Graphical abstract

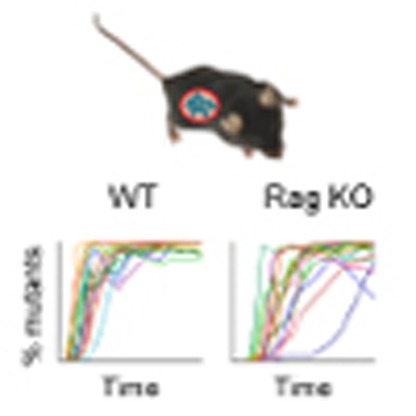

The maintenance of a healthy status in mammals depends on the interaction between their microbiota and the immune system. The partnership between gut commensals and the host is hypothesized to result from millions of years of co-evolution, where the host immune response is tightly regulated to tolerate commensals, which in turn shape its development. Indeed, it recently became clear that specific bacterial species, such as segmented filamentous bacteria^1^, *Clostridia* spp.^2^ and *Bacteroides fragilis*^3^, can promote the expansion of regulatory and pro-inflammatory T cells, promoting immune homeostasis and contributing to restoration of health. Further support for a role of microbes in controlling host physiology comes from faecal transplants, which can affect their weight^4^ and even protect hosts from invasion of pathogens^5^. The patterns of microbiota species composition also differ between healthy and immune-compromised mice lacking an adaptive immune system^6^,^7^, suggestive of the hypothesis of co-evolution between host and gut commensals^8^. However, a direct support for co-evolution requires the study of how host genetics affects evolutionary change within the gut microbial species it carries and vice versa. These issues have been poorly studied, probably partly due to the difficulty in conducting studies that demonstrate the direct influence of microbial evolution on mammalian host evolution. It is, however, easier to address the influence of host genetics on microbial ecology and evolution.

*Escherichia coli*, the most abundant aerobe and one of the first species to colonize the human gut, offers a powerful system to investigate how and to what extent the genetic composition of a commensal changes with time when facing a healthy or a compromised host immune system. For example, in patients with inflammatory bowel diseases, such as Crohn's disease and ulcerative colitis, which are increasing in incidence in the human population and are associated with different mutations in genes with important immune functions, often show an increased abundance of Proteobacteria, namely *E. coli*, which tends to be sampled, in comparison with healthy hosts^9^,^10^.

Using a classical *E. coli* colonization system—the streptomycin-treated mouse–we have recently shown that *E. coli* undergoes rapid adaptation to the intestinal tract^11^. This adaptation was detected through observation of phenotypic sweeps, which occurred across populations of *E. coli* evolving independently in genetically identical immune-competent hosts. Here, we have tested the hypothesis that the process of adaptation of *E. coli* can be altered in immune-compromised mice lacking an adaptive immune system (*Rag2*^−/−^). We show that evolution of *E. coli* is slower in these hosts than in immune-competent animals, due to smaller selective effects of the first beneficial mutations that emerge in these bacteria. We also demonstrate that the strength of natural selection is dependent on the composition of the microbiota, which differs between immune-competent and immune-compromised animals. Furthermore, we describe the genetic targets of *E. coli* adaptation and find adaptive mutations specific to the host genetic background. Finally, the findings that emerging mutations exhibit strong beneficial effects in healthy hosts but substantial antagonistic pleiotropy in immune-deficient mice, support the notion that the adaptive immune system enhances the predictability of adaptive evolution of bacteria comprising the microbiota. Together, these results indicate that not only the microbiotic environment but also the pace and the path of adaptation of a commensal species can be altered by the host immune system.

## Results

### *E. coli* adaptation is slower in immune-compromised mice

**Figure 1:**
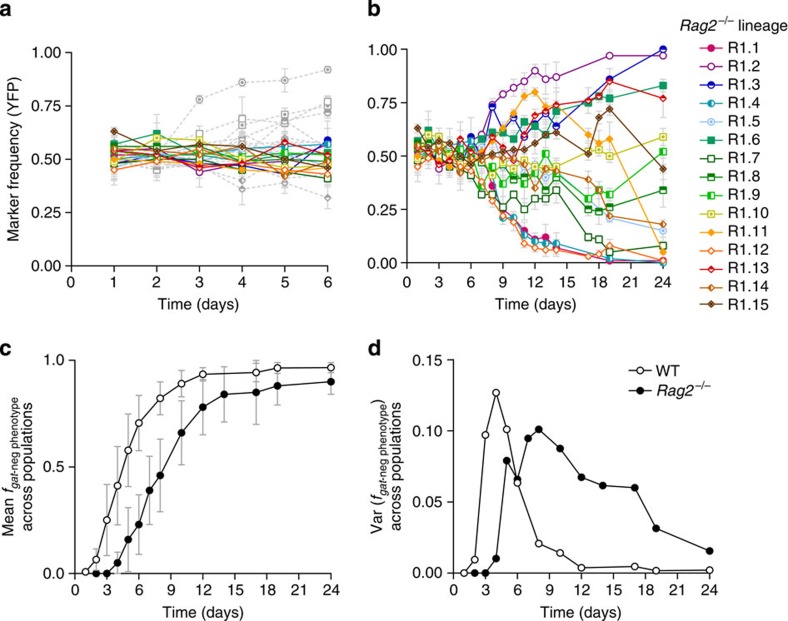
*E. coli* adapts at a slower pace in immune-compromised mice. Dynamics of marker frequency (±2 s.e.m., representing the error associated with the sampling when calculating the frequency of the marker) during the first 6 (**a**) or 24 (**b**) days of adaptation of *E. coli* upon colonization of *Rag2*^−/−^ (coloured) or WT (grey) mice (reproduced from ref. 11 for clarity). (**c**) Average frequency of the *gat*-negative phenotype (±2s.e.m.) over time across WT (white) and *Rag2*^−/−^ (black) animals (*n*=15, for each host genotype) and the variance for the frequency (**d**).

To study *E. coli* adaptation in the gut of *Rag2*^−/−^ mice, we colonized 15 animals (see Methods) with two *E. coli* strains, isogenic except for the presence of a neutral fluorescent marker (cyan fluorescent protein (CFP) and yellow fluorescent protein (YFP)). We first measured the frequency of the neutral marker and its dynamics by daily monitoring of *E. coli* numbers and fluorescence in the faecal content. Much smaller changes in marker frequency were detected in *Rag2*^−/−^ compared with wild-type (WT) mice^11^ during the first 6 days of colonization (Fig. 1a, binomial test *P*=0.03), suggesting a slower rate of adaptation in immune-compromised hosts. This delayed evolutionary change was not due to smaller population size, as similar loads of *E. coli* were recovered from both animals (Supplementary Fig. 1). After this initial period, the marker frequency started to diverge (Fig. 1b) in some *Rag2*^−/−^ animals, as had been observed in WT mice^11^. Some populations showed a signature of a selective sweep, where a single marker steadily increased towards fixation (lines R1.1 and R1.12). Other populations showed the classical signature of clonal interference (R1.11 and R1.15), where clones carrying a given fluorescence first increased in frequency and then were replaced by clones with a different fluorescence, likely due to beneficial mutations with stronger effect arising in the latter. We next asked whether the slower pace of adaptation in *Rag2*^−/−^ mice could be due to a longer time for sweeps to occur. In WT mice, the first adaptive mutations to appear involve changes in genes of the *gat* operon, conferring *E. coli* with a *gat*-negative phenotype^11^. In *Rag2*^−/−^ mice the same phenotype emerged and swept (Supplementary Fig. 2; Fig. 1c), but at a slower pace (z=−25.5, *P*<10^−3^). Though mutations in this operon ultimately reached high frequency in the majority of lineages evolving in *Rag2*^−/−^ mice, they could only be detected after 3 days of adaptation. This contrasts with what was seen in WT mice. This occurred within the same experimental groups (Supplementary Fig. 2, upper panels versus lower panels). Although we detected populations where the increase in frequency of the phenotype was as fast as in WT (Supplementary Fig. 2, lines R1.3 and R1.10), this increase was slower in the majority of the populations. Thus, the *gat*-negative phenotype arose faster (Fig. 1c), and variance in this phenotype was eliminated quicker (Fig. 1d), in WT compared with Rag^−/−^ animals. Both patterns are consistent with faster adaptation under the pressures of an adaptive immune system.

### Duplication time of *E. coli* in the mouse gut

**Figure 2:**
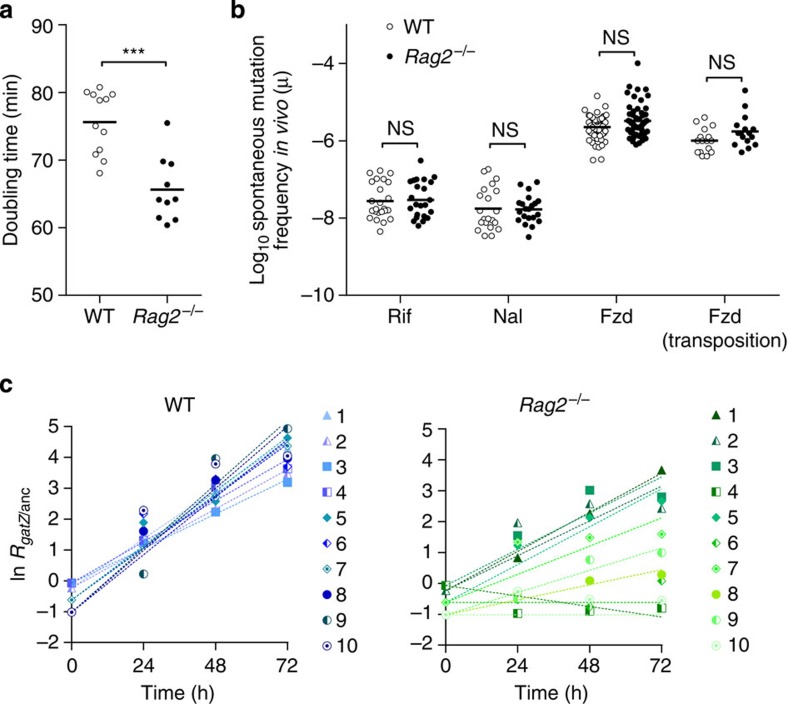
Selection strength, not mutation rate, is responsible for a lower rate of adaptation in immune-compromised mice. (**a**) Higher growth rate of *E. coli* in immune-compromised mice. The doubling time of *E. coli* colonizing the gut of WT or *Rag2*^−/−^ is estimated by the amount of rRNA (see Supplementary Fig. 3). Each symbol corresponds to an average of two independent quantifications made for each mouse (*n*=6) during days 1 and 3 of colonization. The doubling time is significantly lower in *Rag2*^−/−^ than in WT mice, indicating that *E. coli* divides faster in the gut of *Rag2*^−/−^ than in WT animals (Mann–Whitney *U*-test, *W*=111.5, *P*<10^-3^). ***P<0.001. (**b**) Similar frequency of spontaneous mutation in immune-competent and immune-compromised mice: the log_10_ frequency of spontaneous resistance (*μ*) in WT (open circles) and in *Rag2*^−/−^ (full circles) animals is proportional to the mutation rate at mutation-selection balance. Symbols correspond to measurements of mutation frequency over a period of 15 days for rifampicin (*n*=6), nalidixic acid (*n*=6) and furazolidone resistance (*n*=11 and 10). We estimated an average log_10_ *μ*_WT_∼−7.56 and *μ*_*Rag2*_^−/−^∼−7.53 for rifampicin (Mann–Whitney *U*-test, *W*=284.5, *P*=0.95), *μ*_WT_∼−7.76 and *μ*_*Rag2*_^−/−^∼−7.77 for nalidixic acid (Mann–Whitney U-test, *W*=202, *P*=0.65) and *μ*_WT_∼−5.65 and *μ*_*Rag2*_^−/−^∼−5.49 for furazolidone resistances (Mann–Whitney *U*-test, *W*=1613, *P*=0.10). The frequency of spontaneous transpositions corresponds to the fraction of mutations conferring furazolidone resistance, which are caused by insertion of transposable elements: *μ*_TE WT_∼−5.99 and *μ*_TE *Rag2*_^−/−^∼−5.75 (Mann–Whitney *U*-test, *W*=83, *P*=0.09). NS, not significant; *P*>0.05. (**c**) Mean selective advantage of an emerging gat allele is lower in Rag2^−/−^ than in WT mice. Results of short-term competitive fitness experiments (*n*=10) between a *gatZ* mutant and the ancestral *E. coli*. *Rag2*^−/−^ (right panel) or WT (left panel) mice from the same litter are represented by symbols of the same shape (triangles, squares, diamonds or circles). The selective advantage of *gatZ* along the 3 days of competition is inferred from the slope of the linear regression of ln(*gatZ*/anc), (dashed lines, shown for each mouse), which corresponds to the selection coefficient.

We then sought to identify the mechanism responsible for the observed slower adaptive pace in *Rag2*^−/−^ mice. Adaptation rate depends on generation time, population size, mutation rate and the strength of selection on newly adaptive alleles^12^. Population size estimates based on bacterial loads recovered from both groups of animals (Supplementary Fig. 1) suggested that this factor would not play a leading role in the observed differences. To determine the division rate of *E. coli* in the mouse gut, we used *in situ* hybridization with a probe specific for *E. coli* 23S ribosomal RNA (rRNA) to estimate cellular rRNA content, which strongly correlates with bacterial division rate (Supplementary Fig. 3), using an adapted version of a previously described method^13^. We colonized WT and *Rag2*^−/−^ mice with *E. coli* and collected faecal samples at days 1 and 3 after inoculation, when *E. coli* had already reached the same load as observed during the course of the evolution experiment (Supplementary Fig. 1). On the basis of the fluorescence intensity of hybridized *E. coli* cells, we inferred an average duplication time of 66 (±3, 2 s.e.m.) and 76 (±3, 2 s.e.m.) min in *Rag2*^−/−^ and WT mice, respectively (Fig. 2a). As the generation time for *E. coli* was significantly smaller in *Rag2*^−/−^ than in WT mice (Mann–Whitney *U*-test, *W*=111.5, *P*<10^−3^), this parameter also could not explain the different rates of adaptation observed between hosts.

### Estimation of the *E. coli* mutation rate in the mouse gut

Having observed a shorter *E. coli* duplication time in *Rag2*^−/−^ compared with WT animals, we tested the hypothesis that the observed delay in the emergence of the adaptive phenotype in *Rag2*^−/−^ mice was due to a lower spontaneous mutation rate in these hosts. We determined the spontaneous mutation frequency towards resistance to different antibiotics, by measuring the number of clones resistant to rifampicin, nalidixic acid or furazolidone in *E. coli* populations colonizing *Rag2*^−/−^ and WT mice over a period of 15 days (Fig. 2b). Resistance against these antibiotics can be conferred through mutations in specific *E. coli* genes (*rpoB*, *gyrA* and *nfsA*, respectively). Resistance to rifampicin (Rif^R^) and nalidixic acid (Nal^R^) involves mainly point mutations, while resistance to furazolidone (Fzd^R^) can also be acquired by transposition of insertion sequence (IS) elements^14^. Assuming that such resistant alleles are slightly deleterious, and thus kept at mutation-selection balance in a large population, the fraction of resistant mutants is proportional to the mutation rate^12^. We estimated an average log_10_ mutation frequency for Rif^R^ of −7.53 in *Rag2*^−/−^ animals, not significantly different from that in WT mice (Mann–Whitney *U*-test, *W*=284.5, *P*=0.95). A similar result was found for Nal^R^ mutants (−7.76 in WT and −7.76 in *Rag2*^−/−^ mice, Mann–Whitney *U*-test, *W*=202, *P*=0.65) and Fzd^R^ (−5.65 in WT and −5.49 in *Rag2*^−/−^ mice, Mann–Whitney *U*-test, *W*=1613, *P*=0.10). We also measured the *in vivo* frequency of spontaneous resistant mutants to furazolidone, where resistance was achieved through transpositions, therefore providing the first estimate of the *in vivo* spontaneous transposition frequency. This is an important parameter in *E. coli* adaptation to the gut, given that approximately half of the adaptive mutations identified in WT mice were caused by insertion of transposable elements^11^. We estimated an average log_10_ transposition frequency of −5.99 in WT and a similar frequency of −5.75 in *Rag2*^−/−^ mice (Mann–Whitney *U*-test, *W*=83, *P*=0.09). Taken together, these results indicate that both the point-mutation frequency and transposition frequency are similar in immune-compromised and immune-competent animals, suggesting that the slower rate of adaptation observed in the former was not due to an overall decreased mutation rate of *E. coli*.

### Altered selective pressures in immune-compromised mice

We next asked whether the fitness effects of adaptive mutations were different in the gut of *Rag2*^−/−^ and WT mice. We determined the fitness effect (*s*) of a mutant (*gatZ*) harbouring a single beneficial mutation through *in vivo* competition assays against the ancestral *E. coli* (see Methods). In WT mice (Fig. 2c, left panel; Supplementary Table 1), we estimated a mean advantage, per hour (*s*_*gatZ*_), of 0.068 (±0.008, 2 s.e.m.). In contrast, the selective effect of the *gatZ* mutant was smaller in *Rag2*^−/−^ mice (*s*_*gatZ*_=0.03 (±0.01, 2 s.e.m.)) compared with WT (analysis of variance (ANOVA) with Tukey's *post hoc* test, *P*<10^−3^). However, considerably higher variation in the selective effects of the *gatZ* mutation was found in *Rag2*^−/−^ mice (Fig. 2c, right panel; Supplementary Table 1), with a two-tailed test for variance in *s*_*gatZ*_ between *Rag2*^−/−^ and WT being marginally significant (F=0.30, *P*=0.09). The mutation shows antagonistic pleiotropy, that is, in some *Rag2*^−/−^ mice it was advantageous (*Rag2*^−/−^ 1 and 5), while in others it was neutral or slightly deleterious (*Rag2*^−/−^ 4 and 10). We note that variability for the fitness effect of the mutant was observed in mice from the same litter (for example, *Rag2*^−/−^ littermates 3 and 4 or 5 and 6 in Fig. 2c). The strength of natural selection is therefore the principal source of variation in the evolutionary dynamics between the hosts. These results suggest that the selective pressures in the gut of immune-compromised and WT mice are different.

**Figure 3:**
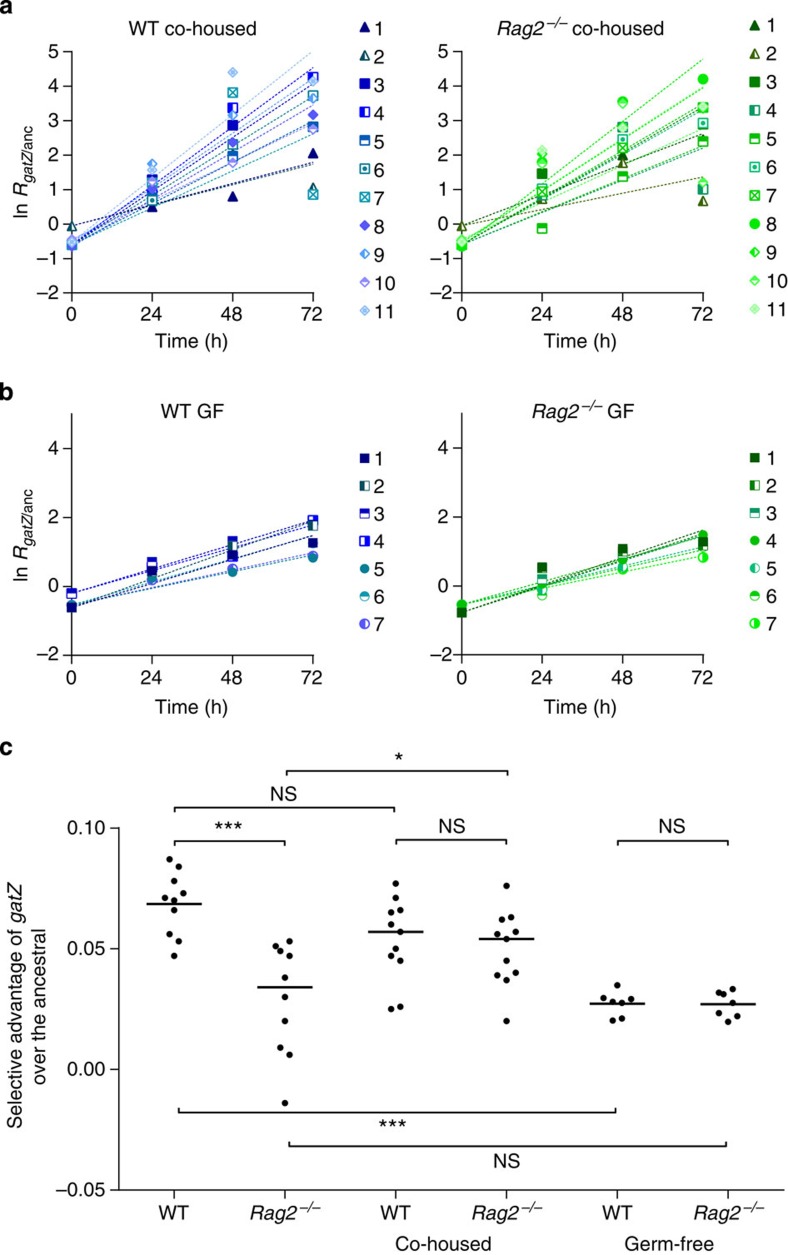
The microbiota influences the selective advantage of adaptive mutations. Competitive fitness experiments of the emerging *gatZ* allele against the ancestral strain in (**a**) WT co-housed with *Rag2*^−/−^ mice (left panel, *n*=11) and *Rag2*^−/−^ co-housed with WT mice (right panel, *n*=11) and in (**b**) GF WT (left panel, *n*=7) and GF *Rag2*^−/−^ (right panel, *n*=7) mice. In **a**, mice co-housed in the same group are represented with the same shape (triangles, squares or diamonds). Advantage of *gatZ* over the ancestral, per hour was calculated as in Fig. 2c. (**c**) Selective advantage of the *gatZ* mutant, per hour, inferred from the slope of the linear regression of ln(*gatZ*/anc), over 3 days of *in vivo* competition against the ancestral in mice either WT, *Rag2*^−/−^, WT co-housed, *Rag2*^−/−^ co-housed, GF WT and GF *Rag2*^−/−^. The competitive advantages differed between *Rag2*^−/−^ and WT (ANOVA with Tukey's *post hoc* test, *P*<10^−3^), but upon co-housing the advantage in *Rag2*^−/−^ mice (co-housed) increased (ANOVA with Tukey's *post hoc* test, *P*=0.02) and became similar to WT animals (co-housed) (ANOVA with Tukey's *post hoc* test, *P*>0.99). The advantage of the WT did not significantly change upon co-housing (ANOVA with Tukey's *post hoc* test, *P*=0.3). In the absence of microbiota (GF), the selective effect of *gatZ* is similar in WT (circles) and *Rag2*^−/−^ (triangles) mice (ANOVA with Tukey's *post hoc* test, *P*>0.99) and smaller than in WT mice harbouring microbiota (ANOVA with Tukey's *post hoc* test, *P*<10^−3^). NS, not significant; *P*>0.05, *P<0.05, ***P<0.001.

Differences in host immune status are associated with differences in microbiota species composition: the microbiota of mice lacking T and B cells has been shown to differ from that in WT controls^7^. To investigate the potential role of the microbiota in driving the changes in adaptation rate observed in this study, we estimated *s*_*gatZ*_ by direct *in vivo* competition against the ancestral in (i) WT and *Rag2*^−/−^ mice previously co-housed for 1 month, a procedure that leads to homogenization of the microbiota between different individuals^15^ (Fig. 3a; Supplementary Table 2), and (ii) germ-free (GF) WT and *Rag2*^−/−^ mice devoid of microbiota (Fig. 3b; Supplementary Table 3). Analysing the full data set (Fig. 3c) including competitions in independently housed WT and *Rag2*^−/−^ animals, revealed a significant overall interaction between host immune status and the microbiota (ANOVA; F_(2,50)_=9.64; *P*<10^−3^), upon the fitness effect of the emerging mutation. In WT animals, co-housing slightly (*s*_*gatZ*_=0.05±0.01) but not significantly (ANOVA with Tukey's *post hoc* test, *P*=0.3, Fig. 3c) decreased the mutant fitness (Fig. 3a, left panel). This advantage was similar to that measured in co-housed *Rag2*^−/−^ (*s*_*gatZ*_=0.050±0.009, ANOVA with Tukey's *post hoc* test, *P*>0.99), which was higher than in independently housed *Rag2*^−/−^ (ANOVA with Tukey's *post hoc* test, *P*=0.02). Another marked effect of the co-housing protocol was a reduction in the variance for the fitness effect of *gatZ* in *Rag2*^−/−^ mice, which became more similar to that observed in WT animals (F-test, F=1.32, *P*=0.67), indicating loss of antagonistic pleiotropy.

Analysis of GF animals provided further evidence of a major role for the microbiota in shaping the selective pressure on the *gatZ* mutant. First, *s*_*gatZ*_ was smaller in GF animals than in microbiota-bearing WT animals (ANOVA with Tukey's *post hoc* test, *P*<10^−3^, Fig. 3c), even though a shorter duplication time was found for *E. coli* in the former (see Supplementary Fig. 4). Second, both the mean and the variance for *s*_*gatZ*_ were similar between GF WT and *Rag2*^−/−^ mice (ANOVA with Tukey's *post hoc* test, *P*>0.99, Fig. 3c; and F-test, F=0.97, *P*=0.97). Finally, irrespective of the functional state of the immune system, the variance for *s*_*gatZ*_ decreased markedly in GF compared with microbiota-harbouring animals (F-test, *P*<10^−3^). Globally, these results confirm the microbiota as a major player modulating the selective effect of beneficial mutations.

**Figure 4:**
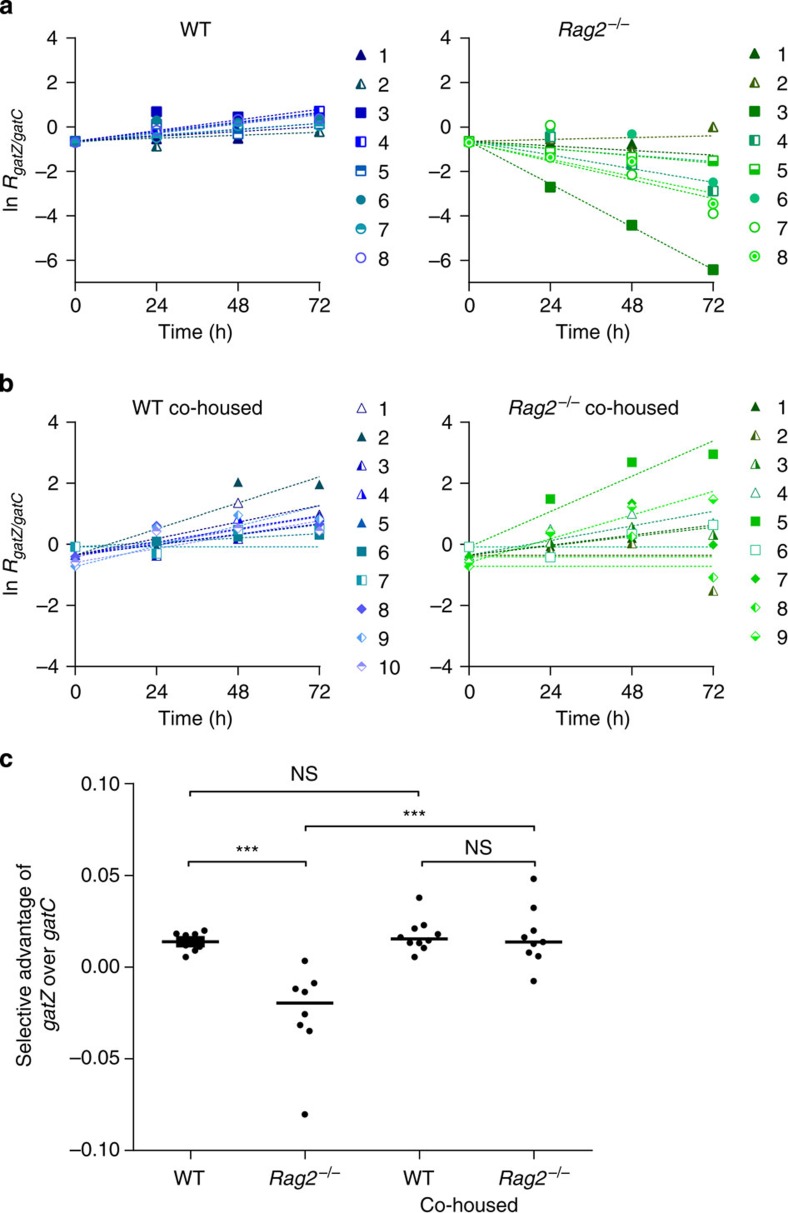
Evidence for antagonistic pleiotropy in immune-compromised mice. Selective effect of *gatZ* allele relative to *gatC* allele in (**a**) WT (left panel) and *Rag2*^−/−^ (right panel), (**b**) WT co-housed with *Rag2*^−/−^ mice (left panel) and *Rag2*^−/−^ co-housed with WT mice (right panel). Competitions were performed in independently housed WT and *Rag2*^−/−^ mice (*n*=8) or in mice co-housed for 1 month (*n*=9). In **a**, mice from the same litter are represented by the same shape symbols (triangles, squares or circles) and in **b**, mice co-housed in the same group are represented with the same shape (triangles, squares or diamonds). (**c**) Comparison of the selective effects of the *gatZ* over *gatC* inferred from the slope of the linear regression of ln(*gatZ*/*gatC*), over 3 days of *in vivo* competition in WT, *Rag2*^−/−^, WT co-housed and *Rag2*^−/−^ co-housed. The mean relative fitness effect differs between *Rag2*^−/−^ and WT (ANOVA with Tukey's *post hoc* test, *P*<10^−3^), but upon co-housing no significant difference was detected between animals (ANOVA with Tukey's *post hoc* test *P*>0.99). Co-housing with WT results in an increase in the mean selective effect of *gatZ* versus *gatC* in immune-compromised mice (ANOVA with Tukey's *post hoc* test, *P*<10^−3^) but not in WT mice (ANOVA with Tukey's *post hoc* test, *P*>0.99). NS, not significant, *P*>0.05, ***P<0.001.

When competing two single *gat* mutants, one carrying an IS insertion in *gatZ* (previously used for the competitions against the ancestral) and the other a single-nucleotide polymorphism (SNP) in *gatC* (see Methods), in independently housed (Fig. 4a; Supplementary Table 4) or co-housed (Fig. 4b; Supplementary Table 5) WT and *Rag2*^−/−^ mice similar results were obtained. Again, we found a significant interaction between the microbiota and the presence of an adaptive immune system (ANOVA; F_(2,31)_=12.9; *P*=0.001). As expected, in WT mice (Fig. 4a, left panel) the mutants were almost neutral (*s*_*gatZvsgatC*_=0.01±0.004). However, in *Rag2*^−/−^ mice (Fig. 4a, right panel) *s*_*gatZvsgatC*_ was on average smaller (−0.03±0.02, ANOVA with Tukey's *post hoc* test, *P*<10^−3^, Fig. 4c). Extensive variability for the fitness effects was observed, with an extreme case in which *gatZ* was found to have a strongly deleterious effect (Fig. 4a, *Rag2*^−/−^ mouse 3). On the other hand, when performing the same competitions in co-housed animals (Fig. 4b), the average *s*_*gatZvsgatC*_ in *Rag2*^−/−^ mice increased (ANOVA with Tukey's *post hoc* test, *P*<10^−3^, Fig. 4c), approximating the value observed in co-housed WT (*s*_*gatZvsgatC*_=0.02±0.01, ANOVA with Tukey's *post hoc* test *P*>0.99, Fig. 4c). Finally, we also detected a significant increase in the variance for *s*_*gatZ*_ when competed against *gatC* in independently housed *Rag2*^−/−^ compared with WT animals (F-test, F=0.04, *P*<10^−3^).

Our combined fitness data provide strong evidence for antagonistic pleiotropy specific to immune-compromised animals, a feature that reduces the predictability of *E. coli* evolution in these hosts. Moreover, our results indicate that the host, the microbiota and their interactions influence the pace of adaptive evolution of *E. coli* to the mouse gut. Together, these findings suggest that a microbiota shaped by the adaptive immune system provides stronger selective pressures upon an individual bacterial species residing within this environment, which arises in the absence of this host component.

### Microbiota characterization of WT and *Rag2* ^−/−^ mice

**Figure 5:**
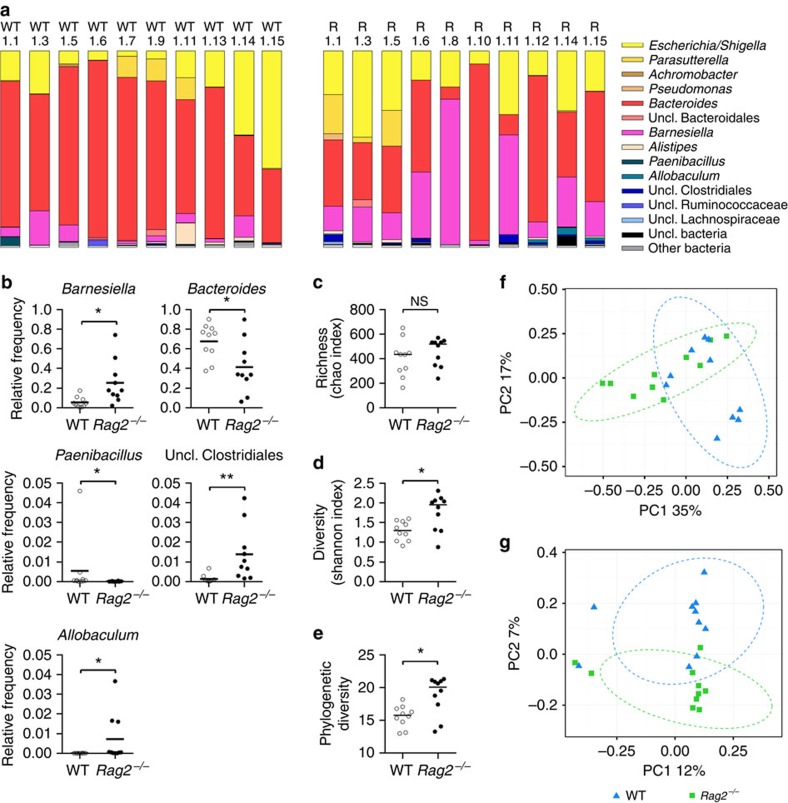
Microbiota composition differs between immune-competent and immune-compromised hosts. (**a**) Microbiota composition at the genus level of 10 WT and 10 *Rag2*^−/−^ mice from the day 3 of the evolution experiment (streptomycin-treated and colonized with *E. coli*). Genera with a relative abundance larger than 1% are displayed. The coloured segments represent the relative frequency of each genus. (**b**) Relative frequencies of genera differentially represented in WT and *Rag2*^−/−^ animals: *Barnesiella* (Mann–Whitney *U*-test with the Benjamini and Hochberg correction, *W*=11, *P*=0.021), *Bacteroides* (Mann–Whitney *U*-test with the Benjamini and Hochberg correction, *W*=18, *P*=0.049), *Paenibacillus* (Mann–Whitney *U*-test with the Benjamini and Hochberg correction, *W*=14.5, *P*=0.029), unclassified Clostridiales (Mann–Whitney *U*-test with the Benjamini and Hochberg correction, *W*=6, *P*=0.0065) and *Allobaculum* (Mann–Whitney *U*-test with the Benjamini and Hochberg correction, *W*=13, *P*=0.025). *P<0.05, **P<0.01. Alpha diversity estimates of microbiota community richness (**c**), diversity (**d**) and phylogenetic diversity (**e**) in WT and *Rag2*^−/−^ animals. Both OTU-based diversity and phylogenetic diversity are increased in *Rag2*^−/−^ animals compared with WT (Mann–Whitney *U*-test, *W*=20, *P*=0.03 and *W*=17, *P*=0.01, respectively). NS, not significant; *P*>0.05, *P<0.05. Principal coordinate analysis (PCoA) of the Bray–Curtis (**f**) and weighted UniFrac (**g**) distance matrices of faecal microbiota of WT and *Rag2*^−/−^ mice. The first two coordinates are shown. Ellipses centred on the averages of the metric distances with a 90% confidence interval for the first two coordinates were drawn on the associated PCoA.

Recent studies have reported differences in microbiota composition between immune-competent and immune-compromised animals^6^,^7^,^16^,^17^,^18^,^19^,^20^. To determine whether changes in the microbiota within WT and *Rag2*^−/−^ raised in our facility were associated with the slower rate and increased variation observed in *E. coli* adaptation in immune-compromised animals, we assessed the composition of this community following high-throughput 16S rRNA gene sequencing of DNA extracted from faecal samples of unmanipulated animals (Supplementary Fig. 5) and from day 3 of the evolution experiment (Fig. 5).

In unmanipulated hosts (*n*=5 for each, see Microbiota analysis in the Methods section), the phylogenetic diversity was significantly higher in WT when compared with *Rag2*^−/−^ animals (Mann–Whitney *U*-test, *W*=23.5, *P*=0.03, Supplementary Fig. 5a). Consistent with this result, PCoA analysis on the phylogeny-based distance of community membership showed a significant difference between genotypes (analysis of molecular variance (AMOVA) on unweighted UniFrac distance, *P*=0.003, Supplementary Fig. 5b). However, at the genus level, difference in abundance was only observed for some low-frequency genera, albeit not statistically significant (Supplementary Fig. 5c). Similarly, operational taxonomic unit (out)-based measures of community richness and diversity revealed only a trend, not significant, for decreased community diversity in *Rag2*^−/−^ (Mann–Whitney *U*-test, *W*=17, *P*=0.4 and *W*=21, *P*=0.1, respectively, Supplementary Fig. 5d,e). PCoA analysis of OTU- and phylogeny-based distances of community structure revealed no significant differences (AMOVA on Bray–Curtis distance, *P*=0.1 and weighted UniFrac distance, *P*=0.4, Supplementary Fig. 5e,f). Importantly, reads identified as *Escherichia/Shigella* could be detected at very small and similar frequencies (maximum 0.03%) in both *Rag2*^−/−^ and WT animals (Mann–Whitney *U*-test, *W*=8.5, *P*=0.46), suggesting that direct competition between our experimental *E. coli* and native *Escherichia*, if any, would be similar in both hosts.

We then analysed 10 WT and 10 *Rag2*^−/−^ animals from day 3 of the evolution experiment, that is, treated with streptomycin and colonized with *E. coli*. The gut microbiota was dominated at the genus level by a few bacterial groups (Fig. 5a), compatible with the strong effect of this treatment, as shown before^21^. In this setting, we identified genera differentially represented in WT and *Rag2*^−/−^ mice (Fig. 5b). Most notably, *Barnesiella, Allobaculum* and an unclassified genus of Clostridiales were more abundant in *Rag2*^−/−^ mice, while *Bacteroides*, and *Paenibacillus* were more prevalent in WT animals. In line with the variance observed when monitoring the *gat* mutant fitness, we detected increased variance in *Rag2*^−/−^ mice for these genera, particularly that of *Barnesiella* (F-test, F=16.3, *P*<10^−3^), for which the relative frequencies ranged between 2 and 74%, while in WT hosts the range was from 0 to 17%. In mice where the rate of increase of the *gat*-negative phenotype (as a proxy for rate of adaptation) was the highest (R1.10) or the lowest (R1.11) (see Supplementary Fig. 2), the microbiota was composed of a small (2%) or large (51%) frequency of *Barnesiella*, respectively.

Although the richness of microbial communities was similar between streptomycin-treated WT and *Rag2*^−/−^ animals (Mann–Whitney *U*-test, *W*=39, *P*=0.4, Fig. 5c), both OTU-based and phylogenetic diversity were increased in the latter (Mann–Whitney *U*-test, *W*=20, *P*=0.03 and *W*=17, *P*=0.01, Fig. 5d,e). Strikingly, when comparing the beta diversity metrics of treated WT and *Rag2*^−/−^ animals, PCoA analysis of Bray–Curtis (OTU-based) and weighted UniFrac (phylogeny-based) distances revealed that the structure of microbial communities significantly differed between the two genotypes (AMOVA on Bray–Curtis distance, *P*=0.01 and weighted UniFrac distance, *P*=0.03, Fig. 5f,g). However, only marginally significant differences were found for the phylogenetic membership (AMOVA on the unweighted UniFrac distance, *P*=0.08, Supplementary Fig. 6).

These results indicate that the microbiota ecosystem experienced by *E. coli* in our experimental setting differs between WT and *Rag2*^−/−^ hosts and between *Rag2*^−/−^ individuals, a feature strikingly reminiscent of the *gat* mutant fitness.

### Genetic targets of adaptation in WT and *Rag2* ^−/−^ mice

**Figure 6:**
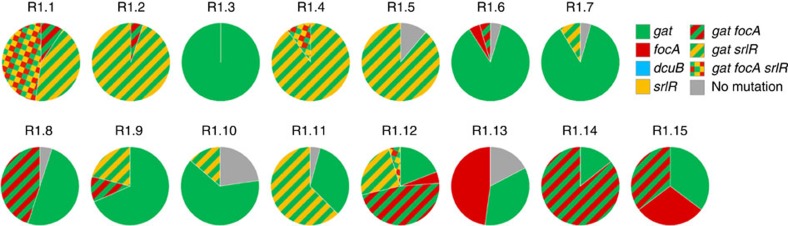
Host genetics influences the genetic basis of *E. coli* adaptation in the gut. Frequencies of haplotypes in *E. coli* populations evolved in the gut of *Rag2*^−/−^ mice for 24 days. Clones (*n*∼20) isolated from the last day of evolution of lineages R1.1 to R1.15 (Fig. 1b) were screened for the presence of mutations previously identified in WT animals: *gat* phenotype (green), insertions in the regulatory region of *focA* (red) and *dcuB* (blue) and SNPs in the coding region of *srlR* (orange). Clones in which none of these mutations were found are shown in grey. Clonal interference is detected by the competition between clones carrying different beneficial mutations (stripes of different colours represent double mutants and clones carrying three mutations are shown as a checkered pattern).

We and others have previously shown that over a 3-week period of evolution, more than one genetic target can be involved in the genetics of *E. coli* adaptation to the mouse gut^11^,^22^,^23^. We therefore thought of determining whether the haplotypic structure of adaptation in *Rag2*^−/−^ mice would be similar to that of WT animals^11^. We sampled clones (∼20) from independent *E. coli* populations recovered from each *Rag2*^−/−^ mouse after 24 days of evolution. Clones were typed for the presence of mutations previously found to be under selection in the gut of WT mice^11^, namely: IS insertions in the intergenic regions of *focA/ycaO* (formate transporter), *dcuB/dcuR* (fumarate transporter) and SNPs in *srlR* (regulator of the sorbitol metabolism). In *Rag2*^−/−^ animals (Fig. 6), IS insertions near *focA/ycaO* as well as SNPs in the *srlR* gene were common (found in 10 and 9 out of 15 mice, respectively). These mutations were segregating at different frequencies within each host: 10–90% for *focA/ycaO* and 10–100% for *srlR* mutations. Strikingly, insertions near *dcuB/dcuR* were not found in the *E. coli* that had evolved in immune-deficient animals (Fig. 6a), although this target was repeatedly identified in clones sampled from WT mice^11^.

The clear occurrence of clonal interference—where clones carrying distinct haplotypes competing for fixation within a population—among *E. coli* evolving in both *Rag2*^−/−^ (Fig. 6) and WT mice^11^, together with the similar rate of spontaneous transposition estimated in these hosts (Fig. 2b), suggests that mutation is non-limiting in the gut of both animals. In turn, this property argues for insertions near *dcuB* to be beneficial in WT, but neutral or even slightly deleterious in *Rag2*^−/−^. To test this hypothesis and identify other targets of adaptation, we performed whole-genome sequencing (WGS) of large population samples (>1,000 clones) that had evolved in each animal. This approach allows the identification of mutations segregating at high frequency, and thus likely to be beneficial, but not those present at low frequency. As expected, WGS (Table 1; Supplementary Tables 6 and 7) revealed the *gat* operon, the *srlR* locus and the intergenic region of *focA/ycaO* as the main targets of adaptation in both hosts. Remarkably, *dcuB* was hit in 7/14 WT and in none of the 15 *Rag2*^−/−^ population tested (binomial test, *P*=10^-3^), confirming the beneficial effects of insertions near this gene in immune-sufficient and not in immune-deficient mice.

The WGS approach also revealed new *bona fide* targets for *E. coli* adaptation to the mouse gut (Table 1). These are targets that occurred in at least two *E. coli* populations evolving in mice living in allopatry. We detected IS insertions, deletions and nonsense mutations in the coding region of *kdgR*, a transcriptional repressor^24^ regulating the metabolism of sugar acids^25^, and insertions of IS elements in the intergenic region of *yjjP* and *yjjQ*, that code, respectively, for an predicted inner membrane protein and a putative transcription factor^26^. We also found an additional target specific to WT mice: *yeaR*, encoding a protein induced in the presence of nitric compounds^27^.

Interestingly, we identified parallel mutational targets detected only in immune-compromised mice (*arcB*, *frlR* and *rimJ*). *arcB* encodes a transmembrane sensor kinase and together with *arcA*, regulates the expression of the Arc modulon in response to changes in oxygen levels^28^. *frlR* codes for a predicted regulator of the fructoselysine operon^29^ that is responsible for the metabolism of fructosamines. *rimJ* encodes an alanine acetyltransferase involved in modification of ribosomal proteins^30^.

## Discussion

We investigated the process of *E. coli* evolution in the gut of immune-compromised mice and compared it with the evolutionary patterns observed for the same ancestral strain in WT mice. We observed a difference in the initial divergence of the neutral marker in immune-compromised compared with immune-competent animals, compatible with a slower rate of evolution in the former. This finding is further supported by a delayed emergence of the adaptive *gat* phenotype, whose dynamics displayed a slower pace and increased variability in its fitness effects among these hosts. In a system of intense clonal interference, as is the case here, the power of the two-marker system in detecting adaptive mutations becomes rapidly reduced. Consequently, the lack of differences in marker dynamics between hosts over longer periods is not interpretable.

Our findings of a higher growth rate of *E. coli* in *Rag2*^−/−^ animals compared with WT is compatible with a weakened control of bacterial growth in immune-compromised hosts, suggesting a more benign environment for *E. coli*. The same explanation may also be valid for the GF mice, where even lower duplications times, regardless of the immune state, were observed. In agreement with this conjecture, the selective effects of the first sweeping beneficial mutations were smaller in immune-compromised hosts, indicating stronger selective pressures in WT mice.

The bulk of our work indicates that the adaptive immune system indirectly increases this selective pressure through the shaping of the microbiota, which in turn interacts with *E. coli*. First, alteration (co-housing) or removal (GF) of the microbiota modulates the selective effects, clearly revealing that this is a major factor controlling the selective pressures in the mouse gut. Second, we could indeed detect differences in the relative abundance of some genera, notably *Barnesiella* spp, between the two host genotypes. Bacteria belonging to this genus have been shown to confer protection against vancomycin-resistant *Enterococcus*, through some undefined direct or indirect mechanism^31^. Our results obtained upon antibiotic treatment in *Rag2*^−/−^ animals, not only show an increase in the mean frequency of *Barnesiella* but also a substantial degree of inter-individual variation, supporting a role for the host adaptive immune system in maintaining coherence in the ecology and evolution of the commensal microbes.

The influence of the host immune system, in particular the adaptive immune system, on the shaping of the gut microbiota has been the focus of recent studies. Among these, the microbiota composition of WT and *Rag1*^−/−^ or *Rag2*^−/−^ mice (both *Rag1* and *Rag2* null mutations result in total ablation of the adaptive immune system) has been compared using 16S analysis^7^,^16^,^17^,^19^. While each study reports differences in the representation of several microbial groups, these are not the same across animal facilities. For example, one work revealed an expansion of anaerobes, especially SFB, in the gut of *Rag2*^−/−^ mice^18^, while others described differences in the abundance of Bifidobacteria and *Clostridium leptum*^19^, or in the phylogenetic community membership such as increased Lachnospiraceae and decreased Porphyromonadacea in *Rag1*^−/−^ mice^17^. None of these specific differences were noticeable in our colonies. In addition, a previous study reported an increased frequency of *Akkermansia* sp in *Rag2*^−/−^ hosts when compared with WT controls^7^. While this genus was represented at notable frequency (5%) in one unmanipulated *Rag2*^−/−^ animal in our study, it was undetectable in the streptomycin-treated mice analysed here, irrespectively of their genotype. However, we detected differences in phylogenetic memberships (unweighted UniFrac) between unmanipulated WT and *Rag2*^−/−^ animals, confirming that the two genotypes do carry different microbial gut compositions. Moreover, our observation of reduced phylogenetic diversity in unmanipulated *Rag2*^−/−^ corroborates other works, where immune-compromised mice, including mutants lacking either or both T and B cells display decreased microbial diversity^6^. A similar finding was reported recently, where the abundance and diversity of B-cell-secreted immunoglobulin-A was correlated with the diversity of the gut microbiota^32^.

More directly relevant to the scope of this work, we found differences in both phylogeny- and OTU-based measures of alpha diversity between streptomycin-treated WT and *Rag2*^−/−^ mice. Together with our findings of distinct community structures (weighted UniFrac and Bray–Curtis distances) between the two genotypes, these results suggest that the pre-existing differences in microbial composition between WT and *Rag2*^−/−^ are exacerbated upon antibiotic treatment. Overall, and in agreement with the majority of previous works, our results indicate that microbial composition differs between immune-competent and -compromised animals, supporting the hypothesis that adaptive immunity plays an important role in the shaping of the microbiota.

The genetic basis of *E. coli* adaptation determined by WGS of evolved populations revealed that most of the mutations were related with bacterial metabolism and respiration, indicating that the main selective pressure was adaptation to the metabolic environment of the intestine. As the microbiota provides key metabolites^33^ and given our findings of different microbial profiles in WT and *Rag2*^−/−^ mice, it is possible that the gut environment of these hosts differs in the availability of different metabolites. In turn, mutations found exclusively or mainly in one host may reflect the specific metabolic composition of that environment. One example, and the most extreme case, is the mutational target *dcuB* found in half of the WT populations but absent in *Rag2*^−/−^ mice. This gene codes for a transporter of C4-dicarboxylic acids such as fumarate and succinate^34^. In the mouse gut, succinate can be produced by *Bacteroides*^35^, a genus that our microbiota analysis revealed to be present at higher relative abundance in WT than in *Rag2*^−/−^ mice. It is possible that differences in the availability of succinate, produced by the microbiota, may have driven the differential selection for mutations in genes related to the uptake and metabolism of this compound in the two groups. The same may be true to *yeaR*, which codes for a protein induced in the presence of nitric compounds^27^, that can be used by *E. coli* to perform anaerobic respiration in the intestine of the host^36^. Similarly, mutations in *frlR*, found in *Rag2*^−/−^ mice, may be related to the levels of fructosamines, compounds that are present in the gut and likely metabolized by bacteria^29^. The mutations identified may lead to inactivation of *frlR* and consequently, to the constitutive expression of the *frl* operon, conferring an advantage for *E. coli* in the gut when nutrients are available at low concentration. Another example is *kdgR*, a transcriptional repressor of the Entner–Doudoroff aldolase-coding *eda* gene^24^. Eda is a key enzyme in the Entner–Doudoroff pathway^25^ responsible for the metabolism of sugar acids, and mutants of this pathway are known to display impaired growth in the mouse gut^22^,^37^,^38^, which is consistent with our finding of selection for mutations inactivating *kdgR*.

Overall, our data reveal that the path and rate of adaptation of commensal bacteria is influenced by the presence of a healthy adaptive immune system, providing experimental evidence to the first corollary of the co-evolution hypothesis between commensal microbiota and the host immune system. Our findings further suggest that the shaping of the gut microbiota composition by the adaptive immune system results in a coherent environment under which *E. coli* evolves along a stronger selective pressure and a more predictable path.

## Methods

### *E. coli* and mouse strains

All bacterial strains used in this study were derived from *Escherichia coli* K-12, strain MG1655 (ref. 39). Strains DM08-YFP and DM09-CFP (MG1655, *galK::YFP/CFP* ampicillin resistant, amp^R^ *(pZ12)*, streptomycin resistant, str^R^ *(rpsl150), ΔlacIZYA*), described elsewhere^11^ were used in the colonization experiments. For *in vivo* fitness assays (see below) two previously described^11^ *gat* mutants of *E. coli* were used: one harbouring an IS in the coding region of *gatZ* (clone 4YFP) and another carrying an insertion of 1 bp in *gatC* (derived from clone 12YFP). These clones were isolated after 24 days of evolution in the WT mouse gut and differed from the ancestral by a single mutation (as confirmed by WGS). Both clones display the *gat*-negative phenotype.

C57BL/6 (WT) and C57BL/6 *Rag2*^−/−^ mice (*Rag2*^−/−^) were used as a model of immune-competent and immune-compromised hosts, respectively. *Rag2*^−/−^ animals carry a null mutation in the *rag* gene essential for the immunoglobulin and T-cell receptor gene recombination and thus lack mature T and B lymphocytes. Six-to-eight-week-old WT and *Rag2*^−/−^ male mice bred at the IGC rodent facility and maintained under strict specific pathogen-free (SPF) conditions (barrier facility with autoclaved caging, food and water) were used for all the experiments, unless otherwise stated. For co-housing experiments, 1-month-old WT and *Rag2*^−/−^ mice (seven animals each) were placed in the same cage for 4 weeks, before *E. coli* colonization. GF C57BL/6 (WT) and C57BL/6 *Rag2*^−/−^ (*Rag2*^−/−^) animals were bred and raised at the IGC gnotobiology facility in dedicated axenic isolators (La Calhene/ORM), an activity partially sponsored by the EU-FP7 InfrafrontierI3-EMMA consortium. Young adults were transferred into sterile ISOcages (Tecniplast) before the competition experiments. All animals were kept in individual cages during both the evolution and the competition experiments.

### Dynamics of adaptation of *E. coli* in the mouse gut

To study *E. coli* adaptation to the gut, we used a streptomycin-treated mouse colonization model^40^. Briefly, SPF-raised mice (see *E. coli* and mouse strains above) were given autoclaved drinking water containing streptomycin (5 g l^−1^) for 1 day. After 4 h of starvation for water and food, the animals were gavaged with 100 μl of a suspension of 10^8^ colony-forming units of a mixture of YFP- and CFP-labelled bacteria (ratio 1:1) grown at 37 °C in brain heart infusion medium to optical density (OD)_600_ of 2. After gavage, the animals were housed in individual cages, and both food and water containing streptomycin were returned to them. Faecal pellets were collected for 24 days, diluted in PBS and plated in Luria Broth (LB) agar. Plates were incubated overnight and the frequencies of CFP- or YFP-labelled bacteria were assessed by counting the fluorescent colonies with the help of a fluorescent stereoscope (SteREO Lumar, Carl Zeiss). A sample of each collected faecal pellet was daily stored in 15% glycerol at −80 °C for further experiments.

For the evolution experiment, groups of five WT and 5 *Rag2*^−/−^ mice were gavaged and followed for 24 days. This procedure was repeated three times, such that a total of 15 mice of each genotype were analysed. While the results of *E. coli* evolution in WT mice were published earlier^11^, experiments in both genotypes were conducted at the same time.

### Dynamics of *gat*-negative phenotype

To investigate the dynamics of appearance and expansion of beneficial mutations in the *gat* operon, we determined the frequency of bacteria unable to metabolize galactitol (*gat*-negative phenotype) within a given bacterial population of the evolution experiment^11^. We plated frozen faecal pellets in Mac Conkey agar supplemented with 1% galactitol and streptomycin (100 μg ml^−1^). Plates were incubated for 20 h at 28 °C. The frequency of galactitol-metabolizing mutants for each time point was estimated by counting the number of white (*gat* mutants) and red colonies in Mac Conkey-galactitol plates.

### Division rate of *E. coli* in the mouse gut

To determine the growth rate of *E. coli* in the mouse gut, we used a modified version of a previously described method^13^ that takes advantage of the correlation reported to occur between growth rate and ribosomal content of bacterial cells. First, we established a calibration curve (see below) that relates the growth rate of *E. coli* populations growing in a given medium and the fluorescent intensity of a probe specific to *E. coli* 23S rRNA (as a measure of ribosomal content). This calibration curve was used to determine the ribosomal content of *E. coli* cells recovered from faecal pellets of WT and *Rag2*^−/−^ mice and infer their growth rates to establish if any differences in the division time of *E. coli* exist in the different hosts.

To obtain a range of growth rates for *E. coli*, we grew *E. coli* strain DM09 (ancestral) in M9 minimal medium supplemented with different carbon sources, which support different growth rates. For the initial inoculum, DM09 was grown for 24 h in 5 ml of LB supplemented with streptomycin at 37 °C with aeration. The culture was washed three times in M9 with no carbon source and diluted to OD 2. 2 μl of a 10^−1^ dilution (∼10^5^ cells) were used to inoculate 198 μl of M9 minimal medium supplemented with one of the following carbon sources: ribose 0.4%, sorbitol 0.4%, xylose 0.4%, fructose 0.4%, maltose 0.4%, arabinose 0.4%, gluconate 0.5% or gluconate 1%. The growth curve assays were performed in a Bioscreen plate incubated in Bioscreen C Microbiology Reader equipment at 37 °C with continuous shaking. ODs at 600 nm were monitored for 48 h. All growth measurements were repeated four times. Specific growth rates were calculated based on the slope of the linear regression of the OD increase over time, during exponential growth. This parameter was then used to calculate the growth rates of *E. coli* in the different media. We then grew new cultures in the same conditions as described above, harvesting the cells in the mid-log phase. The cells were fixed in 4% paraformaldehyde overnight, washed two times in 1 × PBS and stored at −80 °C until use. For the whole-cell hybridization, we used the protocol described by Poulsen *et al*.^13^ with some modifications^41^. Briefly, fixed *E. coli* cells from the *in vitro* cultures were washed once with PBS, once with solution I (35% formamide, 100 mM Tris (pH 7.5), 0.1% SDS and 0.9 M NaCl), and hybridized with a probe specific to *E. coli* 23S rRNA (EC 1531; 5′-CACCGTAGTGCCTCGTCATCA-3′) labelled with the fluorochrome Cy3 (final concentration 2.5 ng μl). The hybridizations were carried for 16 h at 37 °C. The cells were then washed once with solution I and twice with solution II (35% formamide, 100 mM Tris (pH 7.5) and 0.9 M NaCl). Next, the cells were incubated for 15 min at 37 °C, centrifuged and resuspended in 1 × PBS. Cy3 fluorescence was measured by flow cytometry (LSR Fortessa, BD) and median fluorescence analysed with FlowJo v10. For each sample, two hybridizations were performed. The average of median fluorescence intensity of Cy3 was correlated with growth rate for each growth condition, thus enabling a measure of division time of *E. coli*.

WT and *Rag2*^−/−^ mice (*n*=6) were colonized with *E. coli* strain DM09 (ancestral) following the protocol described above for the evolution experiments. Faecal pellets were collected at days 1 and 3 after gavage, homogenized in 1 × PBS and fixed in 4% paraformaldehyde overnight. Following fixation, cells were washed twice in 1 × PBS and stored at −80 °C until use.

Whole-cell hybridization with the probe specific for *E. coli* 23S rRNA was performed according to the protocol described before for the *in vitro* cultures. On the basis of the fluorescence intensity of the probe obtained with the hybridization and the previously obtained calibration curve, we inferred a given duplication time for *E. coli* colonizing WT and *Rag2*^−/−^ mice (see Fig. 2b and Supplementary Fig. 4).

### Comparison of *E. coli* mutation rate between hosts

To estimate the equilibrium frequency for antibiotic resistance clones, we determined the fraction of *E. coli* clones carrying spontaneous resistance to different antibiotics. Slightly deleterious mutations conferring resistance to nalidix acid and rifampicin (point mutations in *gyrA* and *rpoB*, respectively) or furazolidone (mutations in *nfsA*, including IS insertions) are expected to spontaneously occur and be continuously eliminated by purifying selection in *E. coli* populations colonizing the mouse gut, hence resistance alleles are expected to reach a mutation-selection balance and their frequency to reach a stable equilibrium, which is proportional to the mutation rate.

*Rag2*^−/−^ and WT mice were colonized with *E. coli* strain DM08 (ancestral) following the protocol described above for the evolution experiments, and the frequency of spontaneous resistance was determined in faecal pellets collected over 15 days. We tested six animals of each genotype for rifampicin and nalidixic acid resistance and 10 *Rag2*^−/−^ and 11 WT mice for furazolidone resistance. Faecal pellets were collected, homogenized in 1 × PBS and appropriate dilutions plated in LB agar supplemented with streptomycin (100 μg ml^−1^) or LB agar supplemented with streptomycin (100 μg ml^−1^) and nalidixic acid (40 μg ml^−1^), rifampicin (100 μg ml^−1^) or furazolidone (1.25 μg ml^−1^). The frequency of mutants resistant to each antibiotic was calculated as the ratio between the number of antibiotic-resistant clones and the total number of cells in each *E. coli* population. We also estimated the transposition frequency based on the fraction of furazolidone resistance mutants harbouring insertions in *nfsA*.

### *In vivo* competition fitness assays

We measured the relative fitness *in vivo* of an *E. coli* clone harbouring a IS insertion in the *gatZ* gene (clone 4YFP^11^) isolated from faecal samples after 24 days of adaptation to the gut of WT mice. This clone was competed either against the ancestral strain labelled with the opposite fluorescent marker (DM09) (Figs 2 and 3) or against a *gatC* mutant (derived from clone 12YFP)^11^ carrying an insertion of 1 bp in the *gatC* gene (Fig. 4).

The competitions were performed at a ratio of 1:1, over 3 days of co-colonization, following the same procedure described for the evolution experiments in SPF mice; with no streptomycin treatment for competitions carried out in GF mice. We estimated the selective advantage of *gatZ* over the ancestral from the slope of the linear regression of ln(*gatZ*/anc) along time.

### Microbiota analysis

To assess the gut microbiota composition of unmanipulated mice, we analysed faecal samples collected from five independently housed WT and *Rag2*^−/−^ mice belonging to different litters and in two experimental blocks (3+2 mice). These animals were bred and maintained in similar SPF conditions in the same barrier facility (see *E. coli* and mouse strains). With this experimental design, we aimed both to characterize a representative sample of the microbiota composition associated with each host genotype and minimize the cage effects that could bias our analysis.

To characterize the microbiotal context in which *E. coli* was evolving, we analysed faecal samples collected from WT and *Rag2*^−/−^ mice at day 3 of the evolution experiment, that is, streptomycin-treated mice that had been colonized with *E. coli* for 3 days and housed individually. Similarly to unmanipulated animals, these mice were also bred and raised in SPF conditions but treated with streptomycin before and during colonization with *E. coli* (see Dynamics of adaptation of *E. coli* in the mouse gut above). We analysed a total of 10 mice of each genotype, with a minimum of 3 animals from each experimental block (WT: 3+4+3; *Rag2*^−/−^: 3+3+4).

The extraction was performed with a QIAamp DNA Stool Mini Kit (Qiagen), according to the manufacturer's instructions and an additional step of mechanical disruption^21^.

16S rRNA gene amplification and sequencing was carried out at the IGC Genomics Unit. Samples were amplified using primers specific to the V3–V4 region of the 16 s rRNA gene and pair-end sequenced on an Illumina MiSeq Benchtop Sequencer, following Illumina recommendations. Samples were sequenced to a depth of at least 20,000 high-quality sequences. Reads were processed and analysed using mothur^42^ software, following the MiSeq SOP^43^ on the mothur wiki (http://www.mothur.org/wiki/MiSeq_SOP) with some modifications^21^. To minimize possible biases related with variable sequencing depth across samples, a subsampling of 20,000 high-quality reads was performed. Sequences were aligned in accordance with SILVA alignment^44^ and unique sequences were clustered into OTUs using a 97% identity cutoff. A phylogenetic tree was inferred using clearcut^45^ on the 16S rRNA sequence alignment generated by mothur. Alpha and beta measurements of diversity, both OTU- and phylogeny based, were obtained using mothur. The Chao^46^ and Shannon^47^ indices were used to estimate community richness and diversity, respectively. The phylogenetic diversity was calculated as the total of the unique branch length in the phylogenetic tree. As a measure of beta diversity we evaluated the structure of microbial communities, a parameter that takes into account not only the presence and absence of organisms but also their abundance. To assess similarities in community structure OTU- (Bray–Curtis^48^) or phylogeny-based (weighted UniFrac^49^) measures of beta diversity were calculated. Differences in phylogenetic community membership were evaluated using unweighted UniFrac^50^. PCoA on the distance matrices of the beta diversity metrics were performed using Mothur and R software package (http://www.R-project.org).

### Haplotypic structure of *Rag2* ^−/−^ populations

To estimate the *E. coli* haplotypic frequencies evolving in the *Rag2*^−/−^ mice, we analysed ∼20 clones isolated from faecal pellets from the last day of the evolution experiment for each of the evolved lines. These clones were screened for the presence of mutations shown to be adaptive in WT animals^11^: mutations in the *gat* operon, insertions of IS elements in the regulatory regions of *focA* and *dcuB* and point mutations in the coding region of *srlR*. We typed *gat* mutants based on the inability to metabolize galactitol using the procedure described above for the dynamics of the *gat*-negative phenotype. The remaining mutations were identified by target PCR; insertions of IS elements were identified based on the increase in size of the PCR products and Sanger sequencing was used to detect SNPs. PCR reactions were performed in the same conditions and with primers as previously described^11^, but in a total volume of 12.5 μl.

### WGS of *E. coli* populations

To identify the pool of mutations segregating in *E. coli* populations evolved in *Rag2*^−/−^ and WT mice, we performed WGS of a large sample of each population. We isolated more than 1,000 clones from mice faecal pellets from day 24 of evolution, and we extracted the DNA from this mixture of clones following a protocol previously described^11^. The DNA library construction and sequencing was carried out by the IGC genomics facility. Each sample was pair-end sequenced on an Illumina MiSeq Benchtop Sequencer. Standard procedures produced data sets of Illumina paired-end 250-bp read pairs. The mean coverage per sample was between × 100 and × 180 for both WT and *Rag2*^−/−^. Subsampling of 3 million reads was performed to obtain an average of × 100 coverage. Mutations were identified using the BRESEQ pipeline v0.23 with the polymorphism option on (see Supplementary Tables 6 and 7). The default settings were used except for: (a) requiring a minimum coverage of three reads on each strand per polymorphism; (b) elimination of polymorphism predictions occurring in homopolymers of length greater than 3; (c) elimination of polymorphism predictions with significant (*P* value<0.05) strand or base quality score bias. In addition, unassigned new junctions supported by a minimum of two reads in each direction and insertions of IS elements where only one new junction was identified were also accepted. A cutoff of 5% of frequency was used to identify mutations segregating at high frequency in the populations (Supplementary Tables 6 and 7). We defined parallel mutations (Table 1) as mutational events that occurred in a minimum of two animals (either WT or *Rag2*^−/−^) and that reached a minimum frequency of 10% in at least one population.

### Statistical analyses

All statistical analyses were performed in R software: http://www.r-project.org/.

To test for differences in the initial deviation of the fluorescent marker, we used a binomial test that compared the number of WT and *Rag2*^−/−^ lines in which the slope of the linear regression of ln(YFP/CFP) deviated significantly from 0 on the first 6 days of colonization.

We used a linear mixed model on the logarithm of bacterial counts per gram of faeces, with host genotype and time as fixed effects and individuals as random effects to compare the temporal dynamics of bacterial loads between *Rag2*^−/−^ and WT mice. To test whether the bacterial loads over time were affected by host genotype, we contrasted a model with the interaction between these two effects with a model lacking this interaction. Additionally, we also tested if the bacterial loads differed globally between the two genotypes, by comparing models including or excluding the host genotype as a fixed effect.

To assess the influence of host genotype on the temporal dynamics of the *gat*-negative phenotype, we employed a generalized linear mixed model, with host genotype and time as fixed effects and individuals as random effects. The *gat* frequencies were weighted by the number of counted colony-forming units, and the initial frequency was constrained to the same value in WT and *Rag2*^−/−^ dynamics.

Mann–Whitney *U*-tests were used to evaluate differences on the estimated generation time or mutation frequency between WT and *Rag2*^−/−^ mice.

ANOVA was used to investigate the impact of the microbiota and the immune state on the selective advantage of the *gatZ* mutation, followed by Tukey's *post hoc* tests to assess differences between groups. Two-tailed F-tests were used to determine whether the variance for the selective advantage of *gatZ* was increased in *Rag2*^−/−^ compared with WT animals.

Mann–Whitney *U*-tests were used to identify genera differentially represented in WT or *Rag2*^−/−^ mice. The *P* values were adjusted for multiple comparisons using the Benjamini and Hochberg correction. Two-tailed F-tests were used to determine differences in the variance of the genera.

To determine whether the parallel mutations identified by WGS were more represented in one host that in the other, we performed binomial tests.

### Ethics statement

All experiments involving animals were approved by the Institutional Ethics Committee at the Instituto Gulbenkian de Ciência (project no. A009/2010 with approval date 15 October 2010), following the Portuguese legislation (PORT 1005/92), which complies with the European Directive 86/609/EEC of the European Council.

## Additional information

**Accession codes**: Genome sequencing and 16S rRNA gene sequencing data have been deposited in the NCBI Read Archive database with accession code PRJNA297801.

**How to cite this article:** Barroso-Batista, J. *et al*. Adaptive immunity increases the pace and predictability of evolutionary change in commensal gut bacteria. *Nat. Commun*. 6:8945 doi: 10.1038/ncomms9945 (2015).

## Supplementary Material

Supplementary Information Supplementary Figures 1-6 and Supplementary Tables 1-7

## Figures and Tables

**Table 1:** Targets of adaptation differ in *E. coli* evolving in WT and *Rag2* ^−/−^ animals.

Gene	Function	Frequency of mice	
		WT	*Rag2*^−/−^
*gat* Operon	Metabolism of galactitol	100% (±7%)	100% (±7%)
*srlR*	Metabolism of sorbitol	50% (±13%)	60% (±13%)
*focA/ycaO*	Anaerobic respiration (formate transporter)	29% (±12%)	40% (±13%)
***yjjP/yjjQ***	Inner membrane protein	57% (±13%)	13% (±9%)***
*kdgR*	Metabolism of sugar acids	14% (±9%)	13% (±9%)
***dcuB/dcuR***	Anaeobic respiration (fumarate, succinate transporter)	50% (±13%)	0%***
*yeaR*	Metabolism of nitric compounds	14% (±9%)	0%
*arcB*	Regulation of respiration	0%	13% (±9%)
*frlR*	Metabolism of fructosamines	0%	13% (±9%)
*rimJ*	Modification of ribosomal proteins	0%	13% (±9%)

WGS, whole-genome sequencing; WT, wild type.

Frequency (±2 s.e.m.) of WT (*n*=14) or *Rag2*^−/−^ (*n*=15) populations in which parallel mutations at a given locus were found segregating at high frequency (>10%). These loci, identified through WGS of population samples, were observed in at least two populations and thus correspond to *bona fide* targets of beneficial mutations. Mutations whose emergence differs significantly between the two host genetic backgrounds are highlighted in bold, with the level of significance displayed (binomial test ***P<0.001).
