# Supplementary material for: Adaptive immunity increases the pace and predictability of evolutionary change in commensal gut bacteria
Source: Nat Commun. 2015 Nov 30;6:8945. doi: 10.1038/ncomms9945 (PMC4674774; doi:10.1038/ncomms9945)
Supplement: Supplementary Information — Supplementary Figures 1-6 and Supplementary Tables 1-7 [file ncomms9945-s1.pdf]

SUPPLEMENTARY FIGURES

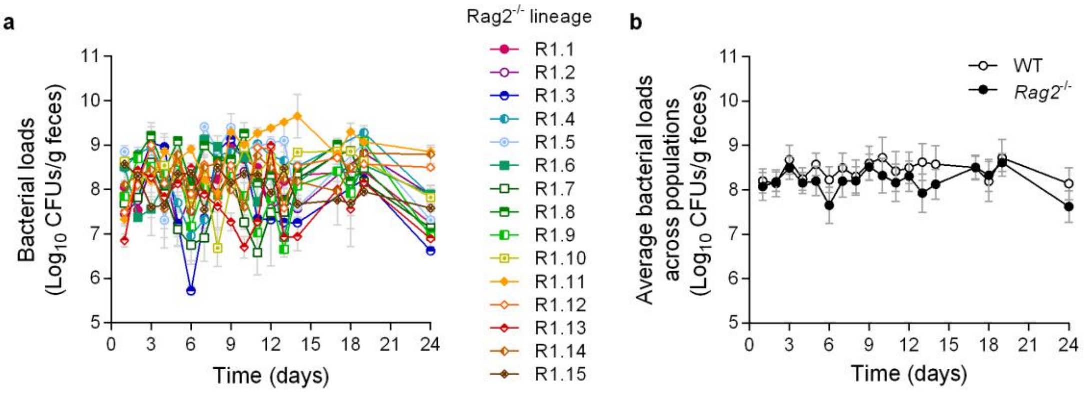

Supplementary Figure 1. Similar *E. coli* load in WT and *Rag2*$^{-/-}$ mice during the evolution experiments

a) *Escherichia coli* colonization of the gut of *Rag2*$^{-/-}$ mice. Bacterial loads per gram of feces (±2 s.e.m) during 24 days of adaptation of *E. coli* to the mouse gut (populations R1.1 to R1.15). **b)** Average bacterial loads (±2 s.e.m) over time in WT (white circles) or *Rag2*$^{-/-}$ (black circles) populations. The host genotype does not influence the temporal dynamics of bacterial loads over time (interaction host genotype and time, ANOVA χ^2_{17}=18.64, p = 0.35) nor the bacterial loads (host genotype, ANOVA χ^2_1=3.35, p = 0.07).

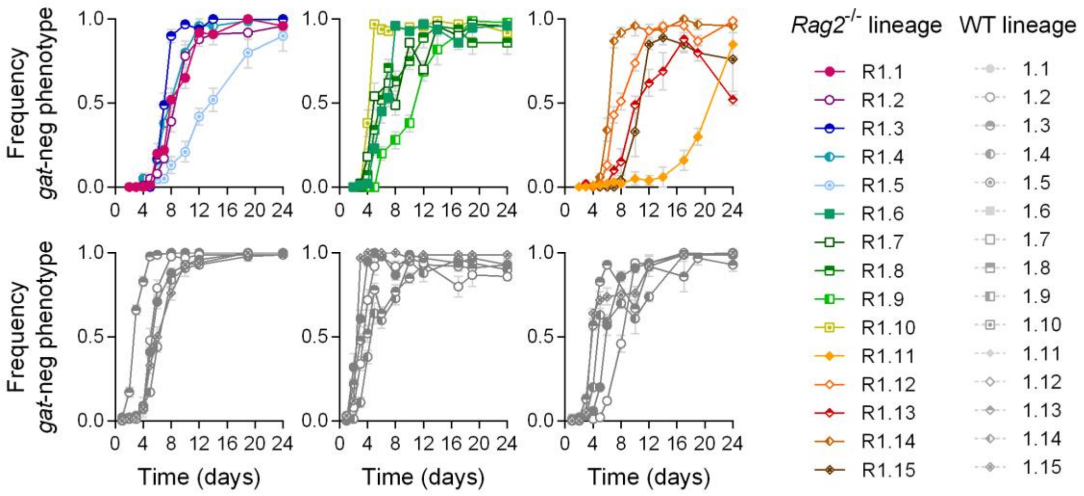

Supplementary Figure 2. Dynamics of the *gat*-negative phenotype

Emergence and expansion of beneficial mutations in the *gat* operon in *Rag2*$^{-/-}$ (upper panels) and WT (lower panels) mice, which cause the increase in frequency of a *gat*-negative phenotype. Each plot displays a group of 5 *Rag2*$^{-/-}$ (upper panels) or WT (lower panels) mice, corresponding to 3 independent experimental blocks.

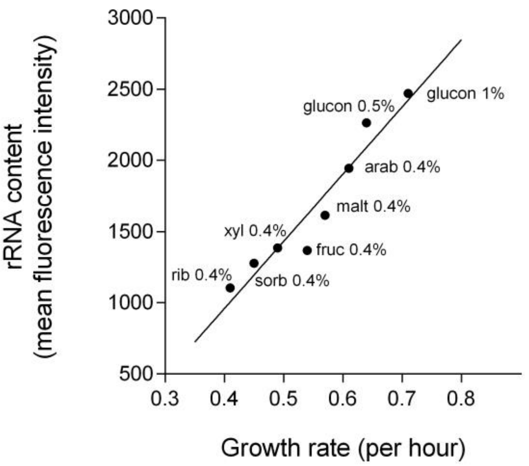

Supplementary Figure 3. Cellular content of ribosomes (cy3 intensity per cell) inferred by whole-cell hybridization

E. coli MG1655 strain DM09-CFP (ancestral) was grown in M9 minimal medium supplemented with (from left to right): ribose 0.4%, sorbitol 0.4%, xylose 0.4%, fructose 0.4%, maltose 0.4%, arabinose 0.4%, gluconate 0.5% and gluconate 1%. Circles represent the average of two hybridization measurements and four growth rates estimates (±2 s.e.m.)

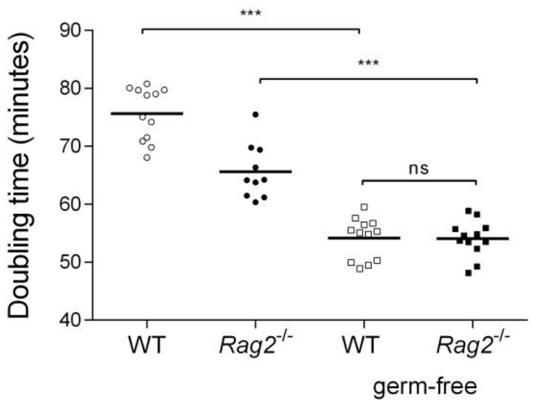

Supplementary Figure 4. Higher growth rate of *E. coli* in GF mice

The doubling time of *E. coli* colonizing the gut of WT or *Rag2*$^{-/-}$ is estimated by the amount of rRNA (see Fig. S3). Each symbol corresponds to an average of 2 independent quantifications made for each mouse (n=6) during day 1 and 3 of colonization. The growth rate is significantly higher in GF compared to microbiota-harboring animals (WT vs WT GF, Mann-Whitney U test with Benjamini and Hochberg correction, W= 144, P<10^{-3} and *Rag2*$^{-/-}$ vs *Rag2*$^{-/-}$ GF, Mann-Whitney U test with Benjamini and Hochberg correction, W=120, P<10^{-3}).

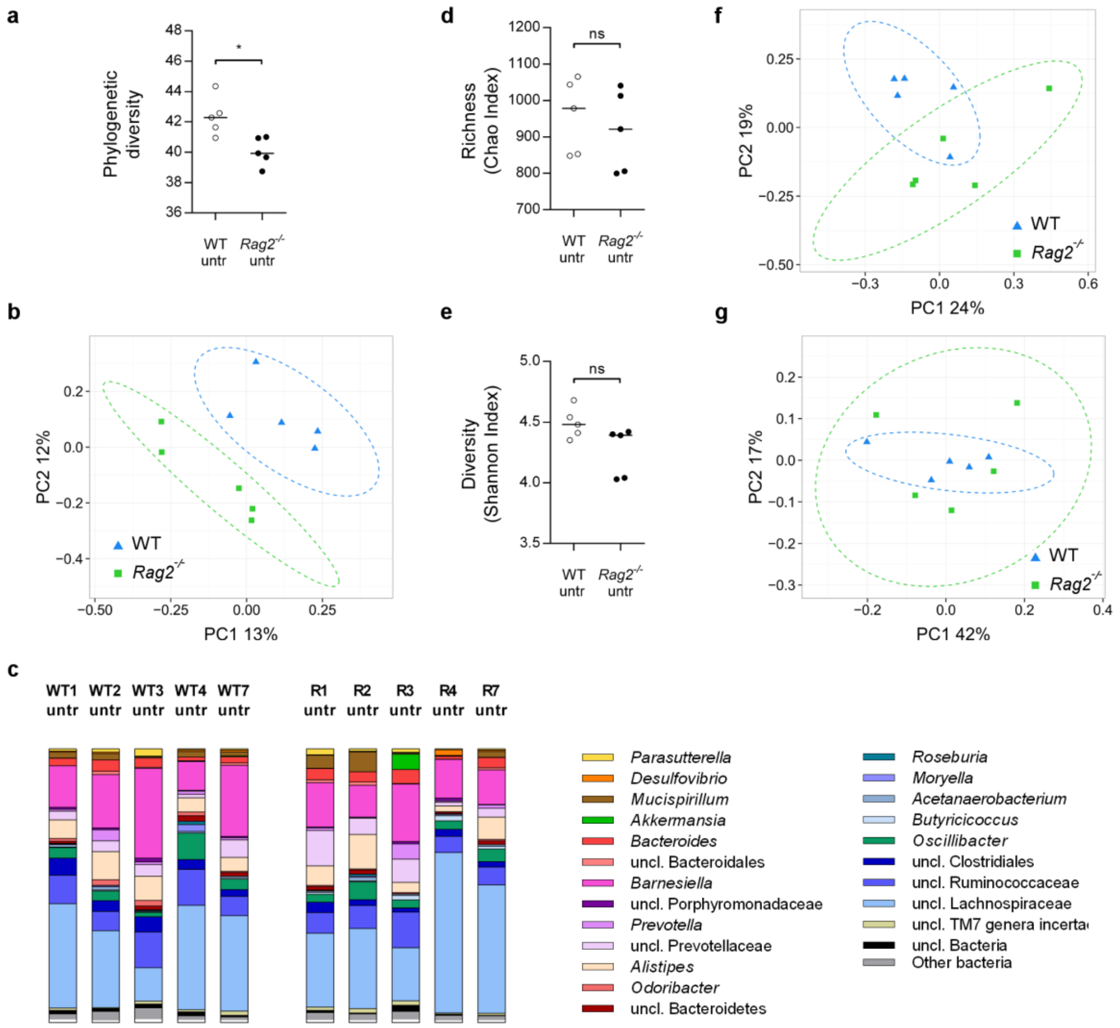

Supplementary Figure 5. Microbiota composition of naïve (unmanipulated) WT and *Rag2*$^{-/-}$ mice.

a) Alpha diversity estimates of microbiota community phylogenetic diversity in WT and *Rag2*$^{-/-}$ animals. Phylogenetic diversity was increased in the WT mice (Mann–Whitney U test, W=23.5, P=0.03). *$P < 0.05$.

b) Principal coordinates analysis (PCoA) of the unweighted UniFrac distance matrices of faecal microbiota of WT and *Rag2*$^{-/-}$ mice. The first two coordinates are shown. Ellipses centered on the averages of the metric distances with a 90% confidence interval for the first two coordinates were drawn on the associated PCoA.

c) Microbiota composition at the genus level of 5 WT and 5 *Rag2*$^{-/-}$ mice before streptomycin treatment and *E. coli* colonization. Genera with a relative abundance

larger than 1% are displayed. The colored segments represent the relative abundance of each genus.

Alpha diversity estimates of microbiota community richness **(d)** and OTU-based diversity **(e)**. Both parameters were similar in *Rag2*$^{-/-}$ and WT animals (Mann–Whitney U test, W=17, P=0.4 and W=21, P=0.1, respectively). Ns (not significant,) $P > 0.05$.

Principal coordinates analysis (PCoA) of the weighted UniFrac **(f)** or Bray Curtis **(g)** distance matrices of faecal microbiota of WT and *Rag2*$^{-/-}$ mice. The first two coordinates are shown. Ellipses centered on the averages of the metric distances with a 90% confidence interval for the first two coordinates were drawn on the associated PCoA.

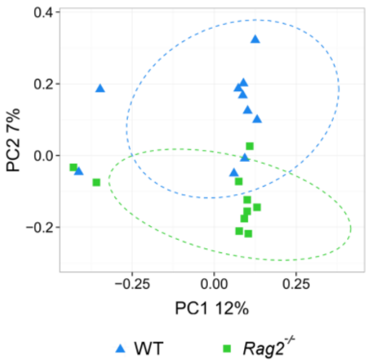

Supplementary Figure 6. Analysis of the phylogenetic membership of WT and *Rag2*$^{-/-}$ mice from the day 3 of the evolution experiment (streptomycin-treated and colonized with *E. coli*).

Principal coordinates analysis (PCoA) of the unweighted UniFrac distance matrices of faecal microbiota of WT and *Rag2*$^{-/-}$ mice. The first two coordinates are shown. Ellipses centered on the averages of the metric distances with a 90% confidence interval for the first two coordinates were drawn on the associated PCoA.

SUPPLEMENTARY TABLES

Supplementary Table 1. In vivo competitions between a *gatZ* mutant and the ancestral strain in WT and *Rag2*$^{-/-}$ mice.

Mice		Time (hours)				*s*
		**0**	**24**	**48**	**72**	
WT	**1**	-0.23	2.32	2.87	4.28	**0.066**
	**2**	-0.23	1.49	2.32	3.47	**0.053**
	**3**	-0.07	1.23	2.23	3.19	**0.047**
	**4**	-0.07	1.31	3.04	3.63	**0.056**
	**5**	-0.62	1.89	2.58	4.63	**0.073**
	**6**	-0.62	2.17	3.30	3.70	**0.070**
	**7**	-0.62	1.66	2.72	4.37	**0.071**
	**8**	-1.02	1.61	3.26	3.97	**0.078**
	**9**	-1.02	0.22	3.96	4.93	**0.087**
	**10**	-1.02	2.28	3.79	4.05	**0.084**
Rag2$^{-/-}$	**1**	-0.23	0.83	2.26	3.67	**0.053**
	**2**	-0.23	1.96	2.58	2.43	**0.047**
	**3**	-0.07	1.55	3.02	2.80	**0.049**
	**4**	-0.07	-0.97	-0.90	-0.80	**-0.014**
	**5**	-0.62	1.23	2.13	2.66	**0.051**
	**6**	-0.62	-0.53	-0.76	0.08	**0.006**
	**7**	-0.62	1.34	1.48	1.59	**0.038**
	**8**	-1.02	-0.31	0.09	0.28	**0.020**
	**9**	-1.02	-0.49	0.76	0.99	**0.030**
	**10**	-1.02	-0.26	-0.52	-0.55	**0.009**

Table displaying the change in ln(*gatZ*/anc), i.e., the natural logarithm of the ratio between the frequencies of the *gatZ* mutant and the ancestral strain, over 3 days (72 hours) of competition in WT and *Rag2*$^{-/-}$ mice (see also Fig. 2c). ***s*** represents the selective advantage of *gatZ* along the three days of competition, inferred from the slope of the linear regression of ln(*gatZ*/anc).

Supplementary Table 2. In vivo competitions between a *gatZ* mutant and the ancestral strain in WT and *Rag2*$^{-/-}$ co-housed mice.

Mice		Time (hours) 0	24	48	72	*s*
WT co-housed	**1**	-0.05	0.50	0.81	2.06	**0.026**
	**2**	-0.05	0.79	2.04	1.06	**0.025**
	**3**	-0.60	1.29	2.87	3.73	**0.065**
	**4**	-0.60	0.87	3.37	4.26	**0.071**
	**5**	-0.60	0.77	1.97	2.82	**0.050**
	**6**	-0.60	0.69	2.31	3.73	**0.060**
	**7**	-0.60	1.20	3.82	0.86	**0.045**
	**8**	-0.65	1.02	2.37	3.17	**0.057**
	**9**	-0.54	1.75	3.16	3.65	**0.066**
	**10**	-0.45	1.24	1.78	2.75	**0.047**
	**11**	-0.52	1.57	4.40	4.14	**0.077**
Rag2$^{-/-}$ co-housed	**1**	-0.05	0.84	2.00	2.45	**0.037**
	**2**	-0.05	0.73	1.78	0.67	**0.020**
	**3**	-0.60	1.46	2.45	2.90	**0.056**
	**4**	-0.60	0.84	2.81	1.01	**0.039**
	**5**	-0.60	-0.13	1.38	2.39	**0.040**
	**6**	-0.60	1.02	2.45	2.93	**0.054**
	**7**	-0.60	0.94	2.21	3.38	**0.057**
	**8**	-0.65	1.79	3.55	4.20	**0.076**
	**9**	-0.54	2.03	2.83	3.35	**0.062**
	**10**	-0.45	1.74	3.49	1.21	**0.045**
	**11**	-0.52	2.14	2.79	3.41	**0.063**

Table displaying the change in ln(*gatZ*/anc), i.e., the natural logarithm of the ratio between the frequencies of the *gatZ* mutant and the ancestral strain, over 3 days (72 hours) of competition in WT and *Rag2*$^{-/-}$ co-housed mice (see also Fig. 3a). ***s*** represents the selective advantage of *gatZ* along the three days of competition, inferred from the slope of the linear regression of ln(*gatZ*/anc).

Supplementary Table 3. In vivo competitions between a *gatZ* mutant and the ancestral strain in WT and *Rag2*$^{-/-}$ germ-free mice.

Mice		Time (hours)				*s*
		**0**	**24**	**48**	**72**	
WT germ-free	**1**	-0.61	0.46	0.92	1.27	**0.029**
	**2**	-0.61	0.43	1.17	1.76	**0.035**
	**3**	-0.20	0.71	1.32	1.79	**0.030**
	**4**	-0.20	0.56	0.87	1.92	**0.028**
	**5**	-0.54	0.24	0.41	0.83	**0.020**
	**6**	-0.54	0.43	0.99	1.25	**0.028**
	**7**	-0.54	0.19	0.51	0.88	**0.021**
Rag2$^{-/-}$ germ-free	**1**	-0.77	0.55	1.08	1.28	**0.033**
	**2**	-0.77	0.52	1.00	1.18	**0.032**
	**3**	-0.77	0.20	0.95	1.25	**0.031**
	**4**	-0.54	0.06	0.76	1.48	**0.028**
	**5**	-0.54	-0.11	0.56	1.19	**0.023**
	**6**	-0.54	-0.27	0.53	1.13	**0.022**
	**7**	-0.54	-0.09	0.49	0.83	**0.020**

Table displaying the change in ln(*gatZ*/anc), i.e., the natural logarithm of the ratio between the frequencies of the *gatZ* mutant and the ancestral strain, over 3 days (72 hours) of competition in WT and *Rag2*$^{-/-}$ germ-free mice (see also Fig. 3b). ***s*** represents the selective advantage of *gatZ* along the three days of competition, inferred from the slope of the linear regression of ln(*gatZ*/anc).

Supplementary Table 4. In vivo competitions between two *gat* mutants (*gatZ* and *gatC*) in WT and *Rag2*$^{-/-}$ mice.

Mice		Time (hours)				*s*
		**0**	**24**	**48**	**72**	
WT	**1**	-0.64	-0.54	-0.51	0.25	**0.009**
	**2**	-0.64	-0.87	-0.24	-0.21	**0.006**
	**3**	-0.64	0.69	0.45	0.42	**0.020**
	**4**	-0.64	-0.11	0.08	0.71	**0.018**
	**5**	-0.64	0.12	-0.32	0.14	**0.011**
	**6**	-0.70	0.30	0.20	0.41	**0.018**
	**7**	-0.70	-0.48	0.05	0.06	**0.012**
	**8**	-0.70	-0.15	0.34	0.37	**0.017**
Rag2$^{-/-}$	**1**	-0.64	-0.71	-0.77	-1.50	**-0.009**
	**2**	-0.64	-0.54	-1.09	0.01	**0.003**
	**3**	-0.64	-2.71	-4.42	-6.42	**-0.080**
	**4**	-0.64	-0.45	-1.69	-2.88	**-0.026**
	**5**	-0.64	-1.13	-1.36	-1.52	**-0.014**
	**6**	-0.70	-0.07	-0.32	-2.48	**-0.012**
	**7**	-0.70	0.08	-2.15	-3.90	**-0.035**
	**8**	-0.70	-1.37	-1.55	-3.45	**-0.032**

Table displaying the change in ln(*gatZ*/*gatC*), i.e., the natural logarithm of the ratio between the frequencies of the *gatZ* and *gatC* mutants, over 3 days (72 hours) of competition in WT and *Rag2*$^{-/-}$ mice (see also Fig. 4a). ***s*** represents the selective advantage of *gatZ* along the three days of competition, inferred from the slope of the linear regression of ln(*gatZ*/anc).

Supplementary Table 5. In vivo competitions between two *gat* mutants (*gatZ* and *gatC*) in WT and *Rag2*$^{-/-}$ co-housed mice.

Mice		Time (hours)				*s*
		**0**	**24**	**48**	**72**	
WT co-housed	**1**	-0.35	-0.17	1.36	0.97	**0.023**
	**2**	-0.35	-0.10	2.04	1.96	**0.038**
	**3**	-0.35	-0.37	0.18	0.90	**0.018**
	**4**	-0.35	0.11	0.84	0.71	**0.016**
	**5**	-0.35	-0.21	0.51	0.58	**0.015**
	**6**	-0.08	0.10	0.25	0.31	**0.006**
	**7**	-0.08	-0.30	0.48	0.50	**0.011**
	**8**	-0.40	0.61	0.54	0.67	**0.013**
	**9**	-0.72	0.55	0.95	0.83	**0.021**
	**10**	-0.59	0.43	0.53	0.44	**0.013**
Rag2$^{-/-}$ co-housed	**1**	-0.35	-0.06	0.21	0.71	**0.014**
	**2**	-0.35	-0.20	0.04	-1.51	**-0.008**
	**3**	-0.35	0.06	0.57	0.32	**0.013**
	**4**	-0.35	0.50	1.00	0.70	**0.020**
	**5**	-0.08	1.49	2.69	2.95	**0.048**
	**6**	-0.08	-0.42	0.34	0.64	**0.008**
	**7**	-0.40	0.37	1.35	-0.01	**0.016**
	**8**	-0.72	0.39	0.26	-1.08	**0.006**
	**9**	-0.59	0.42	1.23	1.48	**0.032**

Table displaying the change in ln(*gatZ*/*gatC*), i.e., the natural logarithm of the ratio between the frequencies of the *gatZ* and *gatC* mutants, over 3 days (72 hours) of competition in WT and *Rag2*$^{-/-}$ co-housed mice (see also Fig. 4b). ***s*** represents the selective advantage of *gatZ* along the three days of competition, inferred from the slope of the linear regression of ln(*gatZ*/anc).

Supplementary Table 6. The number and nature of adaptive events across independently evolved populations of *E. coli* after 24 days of adaptation in WT mice

Population	Genome position	Gene	Mutation	Frequency	
**1.1**	4346885	*dcuB / dcuR*	IS5 +4 bp	77%	
	2173581	*gatZ*	IS1 +9 bp	77%	
	2173490	*gatZ*	IS1 +8 bp	36%	
	3527427	*hslR*	del/dupl 56 bp	6%	*
	4035239	*ileT / ileT/alaT*	del/dupl 11 bp	5%	*
	1269768	*ldrC / chaA*	G→T	7%	
	3873556	*yidX*	T→G	8%	
	4601137	*yjjP / yjjQ*	IS5	5%	¤
	3214187	*yqjH*	del/dupl 82 bp	6%	*
**1.2**	921810	*cspD*	C→T	6%	
	4346885	*dcuB / dcuR*	IS5 +4 bp	48%	
	2172866	*gatA*	IS5 +4 bp	5%	
	2175295	*gatY / fbaB*	IS1 +9 bp	60%	
	2173751	*gatZ*	IS1 +9 bp	30%	
	2173522	*gatZ*	IS1 +9 bp	7%	
	968583	*lpxK/ycaQ / ycaQ*	del/dupl 42 bp	6%	*
	3996061	*uvrD*	del/dupl 39 bp	5%	*
	2180108	*yegW*	IS3	24%	¤
	4601256	*yjjP / yjjQ*	IS5 +4 bp	28%	
**1.3**	4346885	*dcuB / dcuR*	IS5 +4 bp	28%	
	2172866	*gatA*	IS5 +4 bp	7%	
	2175234	*gatY / fbaB*	IS1 +9 bp	13%	
	2175282	*gatY / fbaB*	IS5 +4 bp	5%	
	2173544	*gatZ*	IS1	27%	
	2173511	*gatZ*	IS1	6%	¤
	2173225	*gatZ*	IS1 +9 bp	7%	
	1847931	*sppA*	C→T	8%	
	2711851	*srmB*	del/dupl 79 bp	7%	*
	4601140	*yjjP / yjjQ*	IS5 +12 bp	25%	
	4601120	*yjjP / yjjQ*	IS2 +5 bp	10%	
**1.4**	4346885	*dcuB / dcuR*	IS5 +4 bp	12%	
	2172869	*gatA*	IS5	10%	¤
	2175284	*gatY / fbaB*	IS1 +9 bp	24%	
	2175250	*gatY / fbaB*	IS1 +9 bp	22%	
	2175266	*gatY / fbaB*	IS1	8%	¤
	2173522	*gatZ*	IS1 +9 bp	16%	
	2174014	*gatZ*	IS1 +9 bp	7%	
	2827379	*srlR*	IS1	7%	¤
	285098	*yagG*	del/dupl 22 bp	5%	*
	2143874	*yegE / yehD/yehE*	del/dupl 46 kb	5%	*
	3214187	*yqjH*	del/dupl 82 bp	5%	*
**1.5**	446451	*cyoE*	del/dupl 39 bp	6%	*

	4346885	*dcuB / dcuR*	IS5 +4 bp	31%	
	2172866	*gatA*	IS5 +4 bp	44%	
	2174422	*gatY*	IS1 +9 bp	45%	
	695730	*glnV*	Δ112 bp	6%	
	1907559	*kdgR*	G→A	8%	
	2827493	*srlR*	G→C	39%	
	4601137	*yjjP / yjjQ*	IS5	5%	¤
**1.6**	4346885	*dcuB / dcuR*	IS5 +4 bp	60%	
	3379861	*degQ*	del/dupl 30 bp	6%	*
	2566222	*eutH / eutG*	Δ100 bp	6%	
	2172784	*gatA*	IS1 +9 bp	14%	
	2172866	*gatA*	IS5 +4 bp	7%	
	2172489	*gatB*	IS1 +9 bp	8%	
	2172260	*gatC*	Δ1 bp	27%	
	2175282	*gatY/fbaB*	IS1	5%	¤
	2173533	*gatZ*	IS5 +4 bp	6%	
	2174345	*gatZ / gatY*	IS1 +8 bp	27%	
	1510987	*ydcT*	del/dupl 65 bp	5%	*
**1.7**	953901	*focA / ycaO*	IS5 +4 bp	68%	
	3269213	*garK*	C→T	12%	
	2172535	*gatB*	IS1	10%	¤
	2172265	*gatC*	IS1 +9 bp	7%	
	2175290	*gatY / fbaB*	IS1 +9 bp	66%	
	1907949	*kdgR*	IS5 +4 bp	8%	
	1908116	*kdgR*	IS5 +4 bp	6%	
	1902230	*manZ / kdgR*	Δ 5452 bp	72%	*
	1573764	*yddB*	IS5	7%	¤
**1.8**	2172866	*gatA*	IS5 +4 bp	6%	
	2172221	*gatC*	IS5 +4 bp	49%	
	2172265	*gatC*	IS1 +9 bp	15%	
	2173970	*gatZ*	IS1	10%	¤
	2174027	*gatZ*	IS1 +8 bp	7%	
	2440543	*mnmC*	del/dupl 103 bp	7%	*
	2827492	*srlR*	G→A	28%	
	3634862	*yhiN*	del/dupl 14 bp	7%	*
**1.9**	2880256	*casB*	del/dupl 18 bp	6%	*
	3379861	*degQ*	del/dupl 30 bp	8%	*
	2172633	*gatA*	IS5 +4 bp	29%	
	2172265	*gatC*	IS1 +9 bp	9%	
	2175296	*gatY / fbaB*	IS1 +9 bp	11%	
	2173104	*gatZ*	IS1 +9 bp	25%	
	1434252	*ompN*	del/dupl 16 bp	5%	*
	2827073	*srlR*	A→T	24%	
	1877853	*yeaR*	Δ1 :: IS186 +6 bp :: Δ1	10%	
	2763434	*yfjL / yfjM*	Δ8 bp	8%	
	4601117	*yjjP / yjjQ*	IS5 +4 bp	27%	
	4601148	*yjjP / yjjQ*	IS5 +4 bp	16%	
	4601266	*yjjP / yjjQ*	IS5 +4 bp	6%	
	4601006	*yjjP / yjjQ*	IS2	9%	¤
**1.10**	3379861	*degQ*	del/dupl 30 bp	6%	*
	953892	*focA / ycaO*	IS5 +13 bp	66%	

	2171086	*gatC*	IS1 +8 bp	47%	
	2172265	*gatC*	IS1 +9 bp	15%	
	2174223	*gatZ*	Δ2 bp	33%	
	4601134	*yjjP / yjjQ*	IS5 +4 bp	5%	
**1.11**	2172866	*gatA*	IS5 +4 bp	83%	
	2174150	*gatZ / gatY/fbaB*	Δ1111 bp	14%	*
	2827172	*srlR*	C→A	16%	
	1691840	*uidB*	del/dupl 20 bp	5%	*
	4601110	*yjjP / yjjQ*	IS5 +4 bp	72%	
	4601256	*yjjP / yjjQ*	IS5 +4 bp	7%	
**1.12**	953901	*focA / ycaO*	IS5 +4 bp	20%	
	2172079	*gatC*	.→C	97%	
	2827234	*srlR*	G→A	36%	
	2827117	*srlR*	C→T	6%	
	12154	*yaaI / dnaK*	IS4	47%	¤
**1.13**	4637714	*arcA*	G→T	87%	
	2171150	*gatC*	IS5 +4 bp	93%	
	3265169	*tdcA / tdcR*	IS5 +4 bp	11%	
	572125	*ybcN*	IS2 +5 bp	6%	
**1.14**	4346885	*dcuB / dcuR*	IS5 +4 bp	70%	
	953892	*focA / ycaO*	IS5 +4 bp	58%	
	2172912	*gatA*	IS5 +4 bp	15%	
	2175256	*gatY / fbaB*	IS1 +9 bp	81%	
	2827117	*srlR*	C→T	13%	
	1877853	*yeaR*	IS186 +6 bp	66%	
	4601151	*yjjP / yjjQ*	IS5	7%	¤

Mutations found in *E. coli* populations after adaptation to the gut of WT mice. For intergenic mutations the two flanking genes are listed, otherwise the mutation occurred in the gene coding region. SNPs are represented by an arrow between the ancestral and the evolved nucleotide. The symbol Δ means a deletion event and a + symbol represents an insertion of the nucleotide that follows the symbol. The initials IS denote the abbreviation of insertion sequence element at the indicated position. del/dup indicates that either a deletion or a duplication of the indicated size occurred but it is not possible to distinguish between the two. * indicates that the mutation corresponds to a supported unassigned new junction whereas ¤ denotes a IS insertion where only one new junction was identified.

Supplementary Table 7. The number and nature of adaptive events across independently evolved populations of *E. coli* after 24 days of adaptation in *Rag2*$^{-/-}$ mice

Population	Genome position	Gene	Mutation	Frequency	
**R1.1**	3925453	*asnA*	inv 15 bp	6%	
	953892	*focA / ycaO*	IS5 +4 bp	56%	
	2172791	*gatA*	IS1 +9 bp	8%	
	2172073	*gatC*	IS1 +9 bp	80%	
	2827355	*srlR*	T→C	59%	
	2827528	*srlR*	G→A	13%	
	2827712	*srlR*	+CCCCAA	9%	
	2827388	*srlR*	Δ1 bp	5%	
	3634862	*yhiN*	del/dupl 14 bp	5%	*
	4601143	*yjjP / yjjQ*	IS5	5%	¤
**R1.2**	3925441	*asnA*	del/dupl 39 b	7%	*
	2172008	*gatC*	Δ 7508 bp	90%	
	2827081	*srlR*	C→T	83%	
	3214187	*yqjH*	del/dupl 82 bp	8%	*
**R1.3**	3350864	*arcB*	+AAA	37%	
	3925441	*asnA*	del/dupl 39 b	5%	*
	3502248	*frlR*	IS5 +4 bp	29%	
	3502642	*frlR*	Δ1 bp	7%	
	2172835	*gatA*	IS1 +9 bp	29%	
	2175289	*gatY / fbaB*	IS1	62%	
	2173225	*gatZ*	IS1 +9 bp	8%	
	156651	*yadN*	IS5 +4 bp	10%	
	4592986	*yjjM*	IS1 +9 bp	7%	
	4601157	*yjjP / yjjQ*	IS1 +8 bp	5%	
	2039432	*zinT*	IS5 +4 bp	14%	
**R1.4**	2173491	*gatZ*	IS5 +4 bp	94%	
	2827492	*srlR*	G→A	87%	
**R1.5**	2172972	*gatA*	IS1 +9 bp	84%	
	2752245	*ratB*	del/dupl 23 bp	5%	*
	2827793	*srlR*	T→G	85%	
	3634862	*yhiN*	del/dupl 14 bp	6%	*
**R1.6**	2172774	*gatA*	IS1	6%	¤
	2173014	*gatA*	IS5	5%	¤
	2175284	*gatY / fbaB*	IS1 +9 bp	81%	
	4035239	*ileT / ileT/alaT*	del/dupl 21 bp	7%	*
	932176	*lrp*	G→T	8%	
**R1.7**	3925441	*asnA*	del/dupl 39 b	5%	*
	3496103	*cysG*	del/dupl 5 bp	5%	*
	251816	*dinB*	del/dupl 48 bp	5%	*
	2172211	*gatC*	IS1 Δ4 bp	22%	
	2175244	*gatY / fbaB*	IS1 +9 bp	71%	
	2827492	*srlR*	G→A	43%	

	2891617	*ygcN / gcvP*	del/dupl 153 kb	63%	*
**R1.8**	3925441	*asnA*	del/dupl 39 b	5%	*
	953892	*focA / ycaO*	IS5 +4 bp	63%	
	3502032	*frlD / frlR*	IS4 +12 bp	7%	
	2172866	*gatA*	IS5 +4 bp	51%	
	2175266	*gatY / fbaB*	IS1 +9 bp	16%	
	2173571	*gatZ*	IS1 +9 bp	9%	
	1125212	*rimJ*	IS5 +4 bp	15%	
	761046	*sucB*	del/dupl 67 bp	7%	*
	931615	*trxB / lrp*	IS5 +4 bp	5%	
	3240783	*uxaA*	del/dupl 26 bp	5%	*
	3856228	*yidJ*	del/dupl 23 bp	5%	*
	3152375	*yqhC*	del/dupl 13 bp	6%	*
**R1.9**	4359175	*cadC*	A→T	8%	
	2173093	*gatA*	IS1 +8 bp	5%	
	2173093	*gatZ*	IS1 +9 bp	38%	
	2174199	*gatZ*	G→A	37%	
	1907809	*kdgR*	Δ2 bp	26%	
	2827627	*srlR*	G→A	26%	
	1221585	*ymgD*	C→T	25%	
**R1.10**	3379861	*degQ*	del/dupl 30 bp	5%	*
	628178	*entA*	del/dupl 97 bp	6%	*
	2172912	*gatA*	IS5 +4 bp	45%	
	2172866	*gatA*	IS5 +4 bp	13%	
	2175244	*gatY / fbaB*	IS1 +9 bp	35%	
	1907971	*kdgR*	IS1	12%	¤
	4197319	*nfi*	del/dupl 40 bp	5%	*
	298917	*paoC*	del/dupl 53 bp	7%	*
	3878678	*recF*	del/dupl 62 bp	7%	*
	1125212	*rimJ*	IS5 +4 bp	12%	
	2827490	*srlR*	C→T	21%	
	2030708	*yedJ*	del/dupl 50 bp	6%	*
**R1.11**	2173028	*gatA*	IS1 +9 bp	76%	
	2172073	*gatC*	IS1 +9 bp	6%	
	2173584	gatZ	IS1	*6%*	¤
	2173160	*gatZ*	IS1 +9 bp	6%	
	3325215	*rlmE*	del/dupl 37 bp	5%	*
	2827712	*srlR*	G→A	50%	
	2827492	*srlR*	G→A	13%	
**R1.12**	953901	*focA / ycaO*	IS5 (+) +4 bp	85%	
	1985465	*ftnB / yecJ*	del/dupl 9 bp	5%	*
	2172866	*gatA*	IS5 (+) +4 bp	97%	
	2827184	*srlR*	A→G	36%	
**R1.13**	3350023	*arcB*	T→C	33%	
	953919	*focA / ycaO*	IS1	7%	¤
	2172153	*gatC*	IS1 +9 bp	5%	
	2173559	*gatZ*	IS1 +9 bp	47%	
	3353621	*gltB*	del/dupl 52 bp	5%	*
	1482153	*hrpA*	del/dupl 35 bp	6%	*
	4035239	*ileT / ileT/alaT*	del/dupl 21 bp	6%	*
	2373853	*menC*	IS186	7%	¤

	2317269	*rcsC*	del/dupl 17 bp	5%	*
	2066430	*yoeH*	del/dupl 5 bp	5%	*
**R1.14**	953901	*focA / ycaO*	IS5 +4 bp	87%	
	2172650	*gatA*	IS1 +8 bp	83%	
	2171899	*gatC*	IS1	8%	¤
	1632600	*tfaQ*	del/dupl 72 bp	6%	*
	4368228	*yjeH*	del/dupl 111 bp	7%	*
**R1.15**	3925441	*asnA*	del/dupl 39 b	9%	*
	953901	*focA / ycaO*	IS5 +4 bp	75%	
	2172889	*gatA*	IS1 +8 bp	30%	
	2173012	*gatA*	IS1 +9 bp	10%	
	2171613	*gatC*	IS1 +9 bp	26%	
	3527427	*hslR*	del/dupl 56 bp	8%	*
	2620189	*purM*	G→T	30%	
	4470157	*pyrB*	del/dupl 49 bp	5%	*
	4383948	*yjeO*	Δ1 bp	5%	

Mutations found in *E. coli* populations after adaptation to the gut of *Rag2*$^{-/-}$ mice. See Supplementary Table 1 legend for further details.
